# Forests influence yeast populations vectored by insects into vineyards

**DOI:** 10.3389/fmicb.2022.1039939

**Published:** 2022-11-21

**Authors:** Beatrice Valentini, Francesca Barbero, Luca Pietro Casacci, Anna Luganini, Irene Stefanini

**Affiliations:** Department of Life Sciences and Systems Biology, University of Turin, Turin, Italy

**Keywords:** yeast, social insects, ecology, wine-making, vineyards, wood

## Abstract

**Introduction:**

In the vineyard, yeast communities impact the ripening and fermentation of grapes and are influenced by geographical location, climate, and soil characteristics. Despite the great advancement in our knowledge of the vineyard mycobiota, a key step of the process leading to the definition of the vineyard yeast community is still poorly understood: if geography, climate, and soil influence the mycobiota, potentially through selection, where do the yeast originate from, and how can they reach the vineyard? In this perspective, it is currently acknowledged that forests host several yeast species and that insects, particularly social wasps, can vector and maintain the yeasts known to populate the vineyard. Alas, the conveyance, fostered by insects, of yeasts from the forest to the vineyard has not been proven yet. In this study, we aimed to assess the existence of links between a potential natural source of yeasts (woods), the vectors (social wasps), and the composition of the vineyard mycobiota.

**Methods:**

For this purpose, the mycobiota of wasps caught in six Italian vineyards were analyzed over 2 years through culturomics approaches.

**Results:**

The results clearly indicate that the presence of wooded areas close to vineyards is associated with particular features of the mycobiota vectored by social wasps. Wasps caught in vineyards near wooded areas bear a higher number of yeast cells and higher biodiversity than insects caught in vineyards far from woods. Furthermore, insects caught in vineyards close to woods bear distinctive yeast populations, encompassing species such as *Saccharomyces cerevisiae*.

**Discussion:**

Overall, our work provides fundamental insights into the ecology of the vineyard mycobiota and highlights the need to maintain a vineyard-woodland mosaic landscape, thus preserving the suitable habitat for yeast species relevant to wine-making.

## Introduction

The yeast populations present on grapes in the vineyard affect vine health, growth, and yield (Gilbert et al., 2014) as well as the organoleptic characteristics of the final product (Capozzi et al., 2015; Carrau et al., 2020; Liu et al., 2020). Several studies have shown that the composition of microbial populations changes according to the geographical location of the vineyard (Bokulich et al., 2014; Gilbert et al., 2014; Kioroglou et al., 2019). According to some reports, vineyards’ microbial communities are influenced by geo-climatic elements, such as solar radiation, average (soil) temperature, latitude, longitude, evaporation capacity (Li et al., 2022), maximum temperature, elevation, net precipitation (Chalvantzi et al., 2021), and physicochemical indexes, such as total sugars, polyphenol, total acid, and pH (Li et al., 2022). Despite the acknowledged impact of natural yeast populations on the wine-making process and of the local characteristics of the vineyard on the composition of such populations, we are still far from fully understanding the factors affecting the vineyards’ mycobiota (Griggs et al., 2021). In particular, our current knowledge of the natural sources of yeasts found in the vineyard is limited and we poorly know how not airborne microorganisms (like most yeast species) could reach and populate the vineyard. To spread in the environment, yeasts rely on animal vectors (Stefanini et al., 2012). In this perspective, recent studies have delved into the potential of insects in maintaining and spreading microorganisms, and in particular yeasts, in natural environments (Stefanini, 2018). To date, yeasts have been isolated from at least 104 insect genera belonging to 47 families (Meriggi et al., 2020). Among the insects found to bear yeasts, social wasps of the genera *Polistes*, *Vespa*, and *Vespula* play a pivotal role in the ecology of *Saccharomyces cerevisiae,* a yeast species primarily responsible for must fermentation and thus fundamental in the wine-making process (Stefanini et al., 2012, 2016). Since wasps bear yeast cells characterized by a broad phenotypic and genetic diversity, they do not seem to impose selective pressure on this microorganism (Dapporto et al., 2016). Despite this relevant information on the vectors potentially capable of transporting yeast cells to the vineyards, it is still unclear if and how combinations of abiotic and biotic factors, such as the habitat type surrounding the vineyard, can influence the yeast populations transported by the vectors to the vineyard.

A vineyard-woodland mosaic landscape could not only provide social wasps with a nesting location but also with a broad range of substrates on which these insects could feed (Raveret Richter, 2000). At the same time, a variety of substrates could host a similarly varied range of yeast species, potentially specialized in residing in different niches (Cavalieri et al., 2022; Spurley et al., 2022). Given that forests are inhabited by a broad range of yeast species (Mozzachiodi et al., 2022), it is fair to hypothesize that the presence of woodlands near the vineyard could affect the composition of yeast populations vectored by social insects to the grapes. To test this hypothesis, we investigated the yeast populations vectored by social insects sampled in vineyards close to and far from woods.

## Materials and methods

### Selection and characteristics of sampling areas

Three couples of vineyards, one close and one far from wooded areas, were selected in the provinces of Alessandria and Cuneo (Piedmont, Italy). Considering the known differences observed among vineyard mycobiota in different geographic locations (Bokulich et al., 2016), the three couples of vineyards were chosen in areas distant at least 8 km from each other, with the farthest couple (Area 2) being more than 75 km far from the other two couples (Area 1 and 3; Supplementary Figure S1A). Although our knowledge is limited to a few well-studied insect species, such as honeybees and bumblebees, the mobility of most flying social insects is estimated to occur in a 400 m radius buffer (Rader et al., 2011), and individuals of some *Polistes* species forage on average within 100 m from the nest (Dew and Michener, 1978). Therefore, each couple of vineyards included vineyards having the following characteristics: (i) one close (less than 100 m from the closest edge of the vineyard) and one far (>500 m, farther than the flying radius of social insects) to the closest wooded area; (ii) implementing the organic or biodynamic management (to avoid potential biases due to antifungal or insecticide treatments), (iii) being distant among each other 2 km at most. In the text, we refer to the study vineyards (Vineyards close to Woods) as “VW” and to the Vineyards used as Controls (far from woods) as “VC.” To assess the differences among vineyards, QGIS (QGIS Development Team, 2022) data relative to the selected areas were retrieved from the Geoportale Piemonte^1^ database. We compared land cover categories and pedological and topographic characteristics data among vineyards. Land cover categories included: shrubs/bushes, lawns/gardens, orchards, pasture, vineyard, arable lands, fallow, poplar plantations, Wood (uncharacterized), Wood: black locust, Wood: downy oak, Wood: oak, Wood: deciduous trees, Wood: poplar, sandy soil, small building, industrial building, agricultural building, residential building, streets, artificial water body, or others. Pedologic data were stock and organic Carbon (content % in the 0–30 cm layer of the soils). Topographic characteristics encompassed: slope (degrees), altitude (meters), and Northing and Easting (respectively expressed as sin(exposition) and cos(exposition), where exposition is expressed in radians; Supplementary Figures S1B–E). Differences between the land cover, topographical, and pedological characteristics were evaluated using a Wilcoxon–Mann–Whitney test with false discovery rate (fdr) multiple testing correction.

### Insects collection and dissection

Insects were caught in flight with an entomological net and individually stored alive in sterile 50 ml tubes then taken to the laboratory, where the tubes were kept at 4°C until the insects’ dissection. Three samplings were made for each vineyard: during the harvest 2020 (the 18^th^ of September), spring 2021 (the 4^th^ of May), and harvest 2021 (the 22^nd^ of September). Before dissection, insect species were identified using the keys by Dvořák and Roberts (2006) and Schmid-Egger et al. (2017). In the text, we refer to Insects caught in Vineyards close to Woods as “IVW” and to Insects caught in Vineyards used as Controls (far from woods) as “IVC.” The dissection was performed as previously described (Stefanini et al., 2012, 2016). Briefly, each insect was transferred at −20°C for 20 min, then washed once in sterile water, then in a 1:2 water:bleach solution, and again in fresh sterile water to eliminate residues of bleach, which could hinder the isolation of microorganisms. Then, each wasp was dissected using sterile tweezers and the intestine content (including the stomach/crop and the gut) was dissolved mechanically in sterile water. Considering the different sizes of the insects’ intestines, *Vespa crabro* intestines were dissolved in 200 μl sterile water, whereas *Polistes* spp. and *Vespula* spp. intestines were dissolved in 100 μl sterile water. At this point, an additional 1:5 dilution in water was prepared for each intestine solution, and 100 μl of the obtained solutions were plated onto a rich solid medium (YPD, 2% Agar) supplemented with penicillin (10,000 U/ml) and streptomycin (10 mg/l) to avoid bacterial growth. Plates were incubated at 28°C for 48 h.

### Yeasts’ isolation, identification, and storage

The yeast colonies grown onto YPD were visually inspected and two colonies for each observed morphology were re-isolated onto YPD before identification through PCR-RFLP (Esteve-Zarzoso et al., 1999) based on the interspecific variability of the ITS marker region, including the ITS1 and ITS2 region and the 5.8S rRNA gene. Primer sequences used for amplifying the ITS1-5.8S-ITS2 region were: Primer ITS1 (FW) 5′-GTTTCCGTAGGTGAACTTGC-3′; Primer ITS4 (RV) 5’-TCCTCCGCTTATTGATATGC-3′. Following an initial denaturing step at 95°C for 1 min to activate the GoTaq DNA polymerase (PROMEGA), DNAs were amplified for 35 cycles (95°C for 30 s, 53.6°C for 30 s, and 72°C for 1 min 30 s) plus a final extension at 72°C for 10 min. The PCR products, upon assessment of the size on a 1% agarose gel, were digested with the HaeIII endonuclease for 1 h at 37°C. The digested PCR products were run on 2.5% agarose gel to assess the size of the fragments. The results were compared to those in Esteve-Zarzoso et al.’s (1999) work to identify the species of the yeast isolate. Identifications were confirmed through Sanger sequencing of the ITS1-5.8S-ITS2 region and comparison against the GenBank database using the standard nucleotide BLAST on the NCBI website.^2^ Sequences of the ITS1 regions were deposited in GeneBank; the GeneBank accession IDs are reported in Supplementary Table S4. In the text, we refer to yeasts isolated from the gut of Insects caught in Vineyards close to Wood as “yIVW,” and to yeasts isolated from the gut of Insects caught in Vineyards used as Controls (far from woods) as “yIVC.” Yeast cells were stored in a sterile 40% (w/v) glycerol solution at −80°C.

### Inferring the number of yeast cells per insect intestine

Upon plating of insects’ intestine contents, as described in “Insects collection and dissection”, the CFUs (Colony Forming Units) were determined for each plate as the number of colonies observed on YPD after 48 h at 28°C. The total number of CFUs per insect was calculated by multiplying the CFUs counted on each plate by the dilution factor (please refer to section 3.2). To test the associations of sampling area and time, type of vineyard, and insect subfamily on the number of CFUs (yeast abundance), we performed a model selection using the Akaike Criterion Information (AICc). We computed a generalized linear model with a negative binomial distribution to account for over-dispersion, using the glm.nb function of the MASS R package (Venables and Ripley, 2002). Model selection was performed using the dredge function from the MuMIn package (Bartoń, 2022), starting with a full model that included the type of vineyard, sampling area and time and their interactions, and the separate fixed effect of insect subfamily. We selected equally plausible models with ΔAICc < 4. Differences among yeast abundances found in insects grouped according to the year or season of catching, area of catching, and insect species were assessed using a Wilcoxon–Mann–Whitney test on log10 transformed data (to account for non-normality of data) and on raw data (to delve into the results of the negative binomial model). After correction for multiple testing (false discovery rate, fdr), comparisons with fdr < 0.05 were considered statistically significant.

### Analysis of yeast populations

Upon identification of the yeast isolates, the Wilcoxon–Mann–Whitney test was used to compare the number of yeast species among insects grouped according to the area of sampling, the insect species, and the type of vineyard. A matrix including the presence/absence of yeast species in the analyzed samples was used to calculate the beta diversity with the Jaccard distance using the vegdist function of the vegan R package (Oksanen et al., 2020). Jaccard distances were used to carry out a Principal Coordinate Analysis (PCoA) analysis with the pcoa function of the ape R package and the results were visualized with the biplot.pcoa function of the ape R package (Paradis and Schliep, 2019). The ITS1-5.8S-ITS2 sequences obtained for yeast species identification were aligned using ClustalW2 with the default settings (Sievers et al., 2011) and the resulting Neighbor-Joining phylogenetic tree was used to infer the Unifrac distances among insects’ yeast populations using the distance function of the phyloseq R package (McMurdie and Holmes, 2013). Unifrac distances were used to carry out a PCoA with the ordinate function of the phyloseq R package (McMurdie and Holmes, 2013) and the results were visualized with the ggplot function of the ggplot2 R package (Wickham, 2016). Significant differences among yeast populations in samples grouped according to the relevant variables were assessed through the adonis function of the vegan R package (Oksanen et al., 2020). To evaluate potential correlations between the presence of the identified yeast species and land cover, topographic, and pedological characteristics of the studied vineyards, the frequency of isolation was calculated for each yeast species and vineyard under investigation, as the number of insects bearing the given species divided by the total number of insects caught in the corresponding vineyard. Then, Spearman’s correlations were calculated between the frequency of isolation and the environmental matrix characteristics as described in “Statistical analyses and results.”

### Phenotypic characterization of yeast isolates

To quantify yeasts’ phenotypic characteristics that could be associated with different capabilities of growing in natural (e.g., plants or the wasps’ gut) or must fermentation conditions, the growth of strains representing the isolated yeast species was assessed in multiple media. Some of the environments under investigation are still poorly characterized, especially the wasp intestines (Stefanini, 2018), thus forbidding the accurate *in vitro* modeling of the conditions. Hence, we used some of the growth tests classically performed to characterize yeast species (as reported in Kurtzman et al., 2011), selected as the most likely to be met by yeasts in the studied environments. Arabinose was tested as it is one of the components of biopolymers such as hemicellulose and pectin, hence it is relevant for yeast survival in nature (by using plants as a substrate). Similarly, sucrose was selected as it is widely present in nature, in particular in plant roots, fruits, and nectars. Minimal media (Yeast Nitrogen Base with or without the supplementation of amino acids) were also included in the test as they mimic an environment with limited nutrient resources. Frozen yeasts’ aliquots were pre-inoculated in 96-well plates filled with 200 μl of YPD (1% yeast extract, 2% peptone, 2% D-glucose), and incubated at 28°C for 24 h. Then, 10 μl of the pre-inoculum cultures were inoculated in 200 μl of: (i) YP supplemented with 2% arabinose, (ii) YP supplemented with 2% sucrose, (iii) YNB (0.67% Yeast Nitrogen Base, 2% D-glucose) (iv) YNB supplemented with drop-out without histidine, leucine, tryptophan, and uracil (0.67% YNB, 2% D-glucose, 0.14% drop-out). All the media were sterilized through autoclaving at 121°C and 1 atm for 20 min. Samples were incubated at 28°C and the optical density at 600 nm (OD_600_) was measured after 24 and 48 h. The instrument used for OD_600_ measurement was the GloMax® Explorer Multimode Microplate Reader (Promega). For each experiment, three biological replicates were carried out. To reduce the potential bias due to different relationships between OD and cell number among different yeast species, we normalized the OD_600_ measured for each strain in the tested substrates by dividing it by the OD_600_ measured for the corresponding strain grown in YPD. The resulting ratio was used to compare the growth capabilities among strains through a Wilcoxon–Mann–Whitney test; the growth capabilities were considered significantly different if the fdr-corrected p.value was lower than 0.05. The Wilcoxon–Mann–Whitney test was also performed between strains grouped according to their species, and the same fdr threshold was used to identify significant differences. To further compare the yeast isolates according to their growth capabilities, a PCA was carried out on the normalized values with the prcomp R function (R core team, 2020) and visualized using the fviz_pca_ind and fviz_pca_var functions of the factoextra R package (Kassambara and Mundt, 2020) to plot samples and variables, respectively. The function fviz_nbclust of the factoextra R package (Kassambara and Mundt, 2020) was used with the option FUNcluster = kmeans to determine the best number of clusters grouping the samples distributed according to the PCA. The quantified traits were then compared with a Wilcoxon–Mann–Whitney test among strains grouped according to the inferred clustering.

### Statistical analyses and results

The p-values reported in the text indicate the results of Wilcoxon–Mann–Whitney tests corrected for multiple testing (false discovery rate) unless a different test is clearly indicated. Additional details on the test results (e.g., Mann–Whitney U value) are reported in supplementary materials. The existence of correlations of insect and yeast biodiversity with factors of the environmental matrix (land cover, pedological, and topographical data) was assessed by means of Spearman’s analysis with the rcorr function of the Himsc R package (Harrell, 2022). Correlations R and the fdr-corrected p.values were then visualized using the corrplot function of the corrplot R package (Wei and Simko, 2021).

## Results

### *Vespa crabro*, *Polistes* spp., and *Vespula* spp. visit vineyards both close to and far from wooded areas

**Figure 1:**
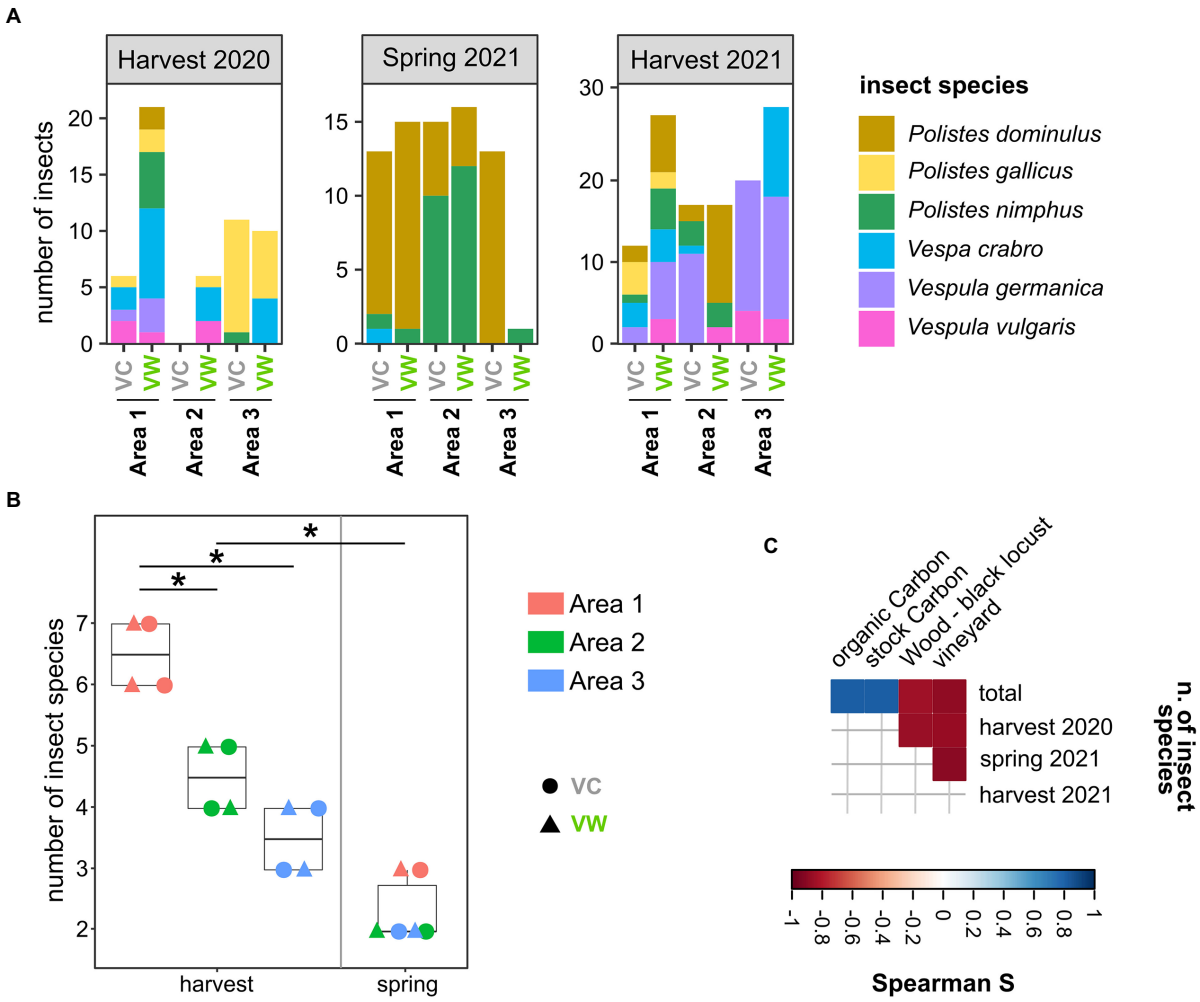
Sampled insects. **(A)** Representation of insect species among the sampled vineyards over the three sampling campaigns. **(B)** Alpha diversity (observed) calculated on insect species caught in the studied areas. Each point corresponds to a different sampling. *Wilcoxon–Mann–Whitney fdr < 0.05. **(C)** Spearman’s correlations between the number of insects caught in this study and the land cover, pedological, and topographical characteristics of the studied areas. Only significant correlations (fdr < 0.05) are shown. VC = Vineyards used as Controls; VW = Vineyards close to Wooded areas.

Wasps and hornets were caught in three couples of organic or biodynamic vineyards located in the provinces of Alessandria (Area 1 and Area 3) and Cuneo (Area 2; Piedmont, Italy), for a total of six vineyards (Supplementary Figure S1; Supplementary Table S1). Each couple encompassed a Vineyard close to a Wooded area (hereinafter named “VW”) and a control vineyard (far from wooded areas, hereinafter named “VC”). The selected vineyards did not show significant differences in pedological, topographical, and land cover characteristics (Supplementary Figures S1B–E). At least 10 adult *Vespa crabro* and *Polistes* spp. wasps were captured in each analyzed vineyard during three sampling events (the harvest 2020, spring 2021, and harvest 2021), resulting in a total of 237 insects. The frequency of several species changed according to the season: the spring sampling provided the lowest number of captured insect species, with a prevalence of wasps of the genus *Polistes* (Figure 1A). Conversely, several additional insect species, also including *Vespa crabro* and *Vespula* spp., were caught in the two harvesting samplings, which showed a significantly higher number of insect species compared to the spring sampling (*p* = 0.001, Figure 1B). This discrepancy can be ascribed to the fact that the number of individuals in *Vespa crabro* and *Vespula* spp. nests increases in late August–September, thus later than the time of the spring sampling (May; Stefanini et al., 2012), hence reducing the chance of sampling insects of these species. In addition, Area 1, one of the two areas located in the province of Alessandria, showed a higher species richness than Areas 2 and 3 (fdr < 0.05; Figure 1B). Spearman’s analysis highlighted that the number of insect species was positively correlated with the amount of stock and organic carbon in the soil (*r* = 0.812 fdr = 0.049 for both) and negatively correlated with the percentage of the area surrounding the vineyards dedicated to other vineyards (*r* = −0.886 fdr = 0.019; Figure 1C). The percentage of the area dedicated to vineyards was also negatively correlated with the number of insect species caught in the harvesting 2020 (*r* = −0.870 fdr = 0.025) and spring 2021 (*r* = −0.899 fdr = 0.015) samplings (Figure 1C). The number of insect species caught in harvest 2021 was negatively correlated with the percentage of the area surrounding the vineyards dedicated to woods populated with black locusts (*r* = −0.857 fdr = 0.029) **(** Figure 1C).

### Insects caught in vineyards close to wooded areas bear a higher abundance and diversity of yeasts than insects caught in control vineyards

**Figure 2:**
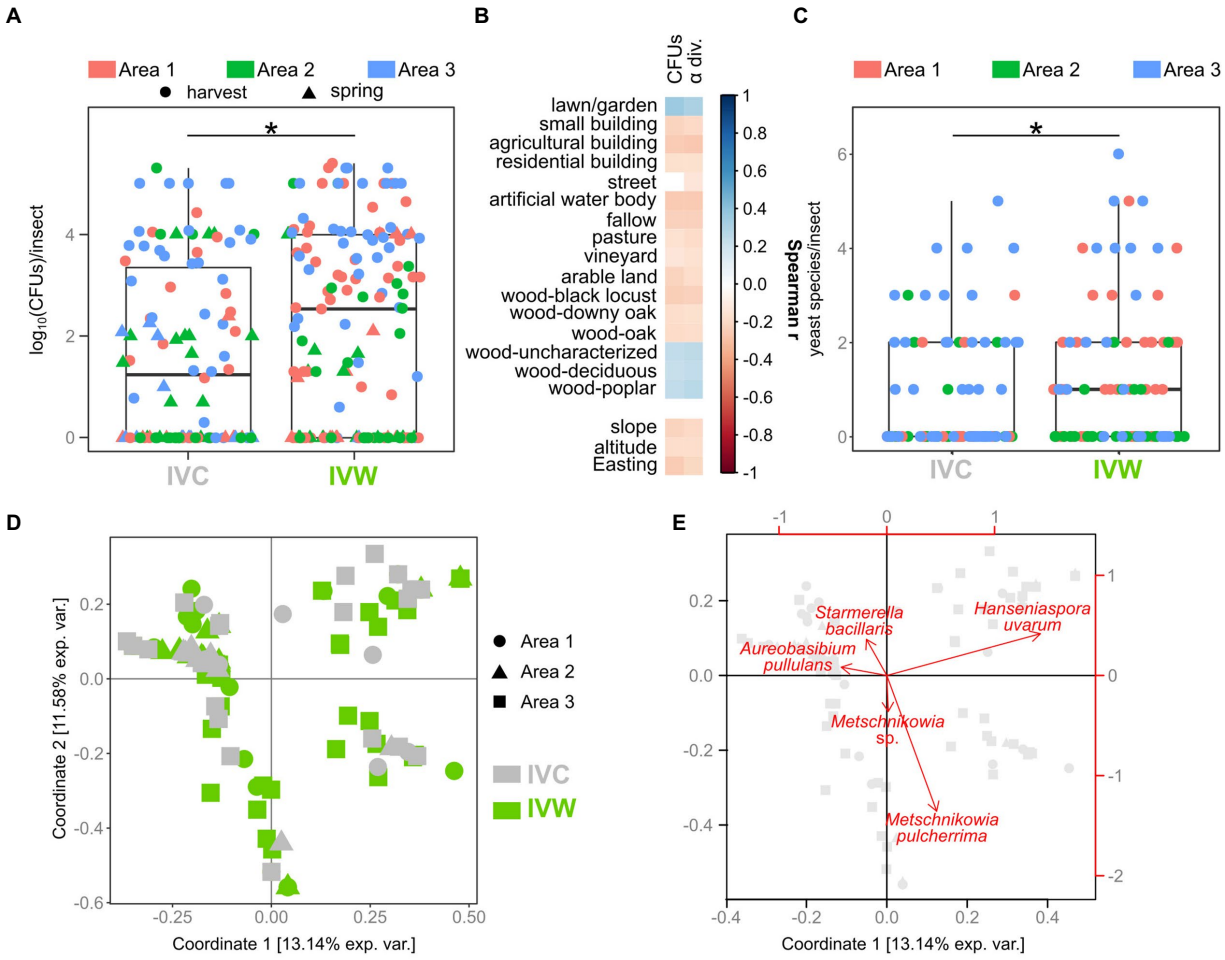
Comparison of abundance, biodiversity, and composition of yeast communities. **(A)** Comparison of yeast abundances in insects caught in vineyards near (VW) and far from (VC) wood. **(B)** Spearman’s correlations between the number of yeast CFUs and alpha diversity found in the studied insects and the land cover, pedological, and topographical characteristics of the study areas **(C)** Comparison of yeast alpha diversity of insects caught in VW and VC. **(D)** Beta diversity of yeast populations in insects caught in VW and VC. Visualization of the first two coordinates of the PCoA based on Jaccard distances calculated on yeast presence/absence data. **(E)** Projection of the most relevant variables (yeast species) on the first two coordinates of the PCoA based on Jaccard distances. Gray symbols indicate the position of samples, as shown in panel (**D)**. * Wilcoxon–Mann–Whitney fdr < 0.05. IVC = Insects caught in Vineyards used as Controls; IVW = Insects caught in Vineyards close to Woods.

Woodlands and anthropogenic areas surrounding the vineyard provide different substrates to insects, thus potentially influencing both the composition and the abundance of yeast gut populations. Hence, we evaluated if the number of yeast cells in the intestine of insects differed between insects caught in vineyards close to (“IVW”) or far from wooded areas (“IVC”) by assessing the number of yeast CFUs (yeast abundance) obtained through culturomics. The yeast abundance was significantly different between IVC and IVW, with the latter bearing more yeast cells than the insects in control vineyards (*p* = 0.014, Figure 2A). The best Negative Binomial Generalized Linear Model explaining yeast abundance included the type of vineyard (wood and control), the sampling time, and the interaction between these two factors (AIC 3317.3; Table 1). It is worth mentioning that the model did not highlight differences between the abundance of yeasts in insects caught in harvesting 2020 and 2021, hence indicating that the vintage has a minor impact than the environmental matrix and season on the number of yeast cells transported by insects (Table 1). Furthermore, significant differences in yeast abundances were observed among insects’ intestines grouped according to the area of sampling (Area 1, Area 2, and Area 3), with Area 3 showing a higher yeast abundance than Area 1 and Area 2 (fdr < 0.05) but not according to the year of sampling (Supplementary Figures S2A,B). The number of yeast CFUs found in wasps’ guts was positively correlated with the percentage of the area surrounding the vineyards dedicated to lawns or gardens, woods composed of deciduous trees or poplars, and uncharacterized woods and negatively correlated with the slope, altitude, and Easting of vineyards, and the percentage of the area surrounding the vineyards dedicated to small, agricultural, and residential buildings, fallow, pasture, vineyards, plantation, woods composed by black locusts, downy oaks, and oaks (Spearman fdr < 0.05, Figure 2B). Vespinae bore more yeast cells compared to Polistinae; *Polistes gallicus* and *P. dominulus* bore fewer yeasts than *Vespa crabro*, *Vespula germanica*, and *Vespula vulgaris* (fdr < 0.05; Supplementary Figure S2C), and insects caught in the harvest samplings bore more yeasts than insects caught in the spring sampling (fdr = 5.849e^−06^, Supplementary Figure S2D).

**Table 1:** Estimated coefficients (*β*^) for the fixed-effect terms in the model with the lowest AIC (3317.3) relating yeast abundance to tested variables.

	*β*^	SE	*p*
Harvest 2020	9.9428	0.5351	<2e-16
Spring 2021	0.1569	0.6553	0.002241
Vineyard type (VC vs. VW)	−4.3266	0.9538	5.72e-06
Harvest2021*control	4.0001	1.1265	0.000384
Spring2021*control	3.4207	1.2244	0.005210

Only significant factor levels are reported. VC = Vineyards used as Controls; VW = Vineyards close to Wooded areas.

Overall, 468 strains belonging to 65 different yeast species were isolated from the analyzed insect intestines (Supplementary Table S3). Not every insect bore yeasts, and a significant relationship between the presence/absence of yeasts and the type of vineyard (Chi-square test, *F* = 10.382, *p* = 0.013) was observed (Supplementary Figure S3). A significant relationship was also found between the yeast presence and the period of sampling (harvest 2020, spring 2021, harvest 2021; Chi-square test, *F* = 12.311, *p* = 0.001) and the sampled area (Chi-square test, *F* = 9.598, *p* = 0.008; Supplementary Figure S3). The alpha diversity of yeast populations (number of yeast species per insect) was significantly different between insects sampled in vineyards close to wooded areas and control vineyards (fdr = 0.018, Figure 2C). The alpha diversity of yeast populations also differed among the three sampling areas, and the insects caught in spring 2021 showed fewer yeast species compared to the insects caught in the other sampling events (fdr < 0.05; Supplementary Figure S4). Spearman’s analysis showed the same correlations between yeast alpha diversity and topographical, pedological, and land cover information as the ones observed between these environmental features and the number of yeast CFUs (Figure 2B). Concerning the comparison of insect species, regardless of the type of vineyard in which they were caught (close to Woods, VW, or used as Controls, VC), *Vespa crabro* showed more yeast species compared to any other insect species besides *Vespula vulgaris*, which, in turn, bore more yeast species compared to *P. dominulus* (fdr < 0.05, Supplementary Figure S4).

Beta diversity analysis (Jaccard distances) showed that the composition of yeast populations of insects caught in vineyards close to woods and in control vineyards significantly differed (permANOVA Df = 5\u00B0F = 2.31, *p* = 0.001, Figure 2D). *Metschnikowia pulcherrima*, *Hanseniaspora uvarum*, *Starmerella stellata*, and *Candida* sp. were the yeast species mostly associated with the first two coordinates of the PCoA carried out on Jaccard distances (Figure 2E). Significant differences among yeast populations were observed when grouping samples according to area and time of sampling, as well as to the insect species (Supplementary Figure S5). Moreover, significant differences were also observed among yeast populations in IVW and IVC guts when considering Unifrac distances, based on the phylogenetic relationships among the yeast species composing the community (Supplementary Figure S6).

### Distinctive yeast species are found in insects caught in vineyards either close or far from woods

**Figure 3:**
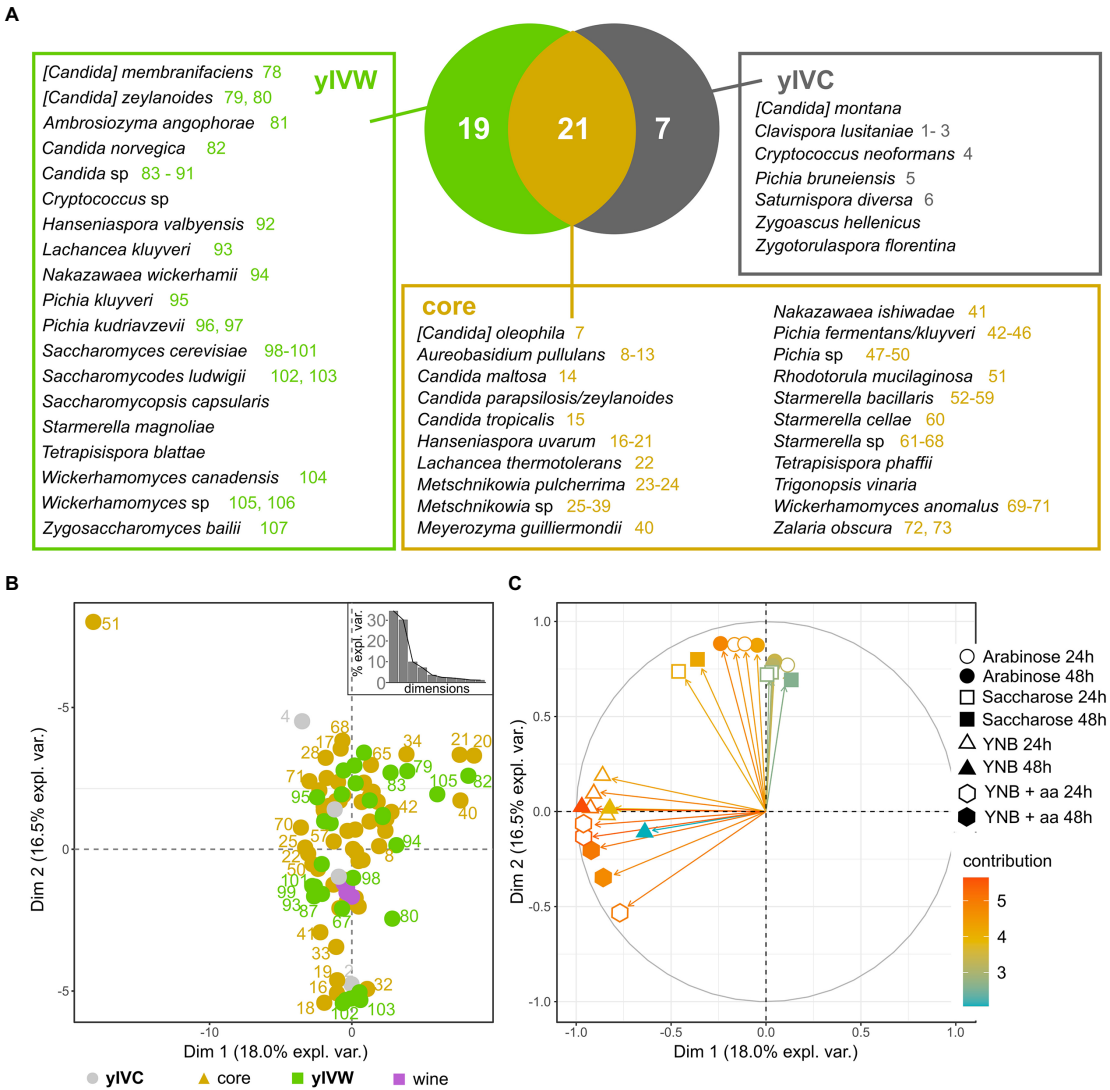
Yeast species isolated from the intestines of investigated insects. **(A)** Venn diagram showing the distribution of yeast species between insects caught from vineyards close to wooded areas (VW) and control vineyards (VC). The yIVW group includes species found only in wood insects, the yIVC group includes species found only in VC insects, and the core group encompasses yeast species found in insects caught in both VC and VW. Colored numbers report the strain IDs indicated in panel B, with the color indicating the group of the yeast species (yIVW, yIVC, or core yeast species). The species without the colored number were not phenotypically characterized. **(B)** The first two components of the PCA performed on distances calculated on the quantified phenotypes of strains representing the identified species and **(C)** projection of the variables according to their relevance in the determination of the variance over the first two PCA components. Each variable includes the quantification of the growth of each tested strain in the conditions reported in the legend. Three independent biological replicates were carried out, used in the PCA analysis, and reported in the plot.

We then delved into the distribution of yeast species in the investigated insect intestines and observed that 21 yeast species were shared by insects caught in vineyards close and far from woods (“core”), 19 species were found only in the gut of wasps caught in vineyards close to wooded areas (yIVW), and 10 yeast species characterized insects caught in control vineyards (yIVC, Figure 3A). *Saccharomyces cerevisiae*, the species commonly responsible for spontaneous alcoholic fermentation and previously found in wasps’ and hornets’ guts (Stefanini et al., 2012), was isolated only from wood insects, whereas the human pathogen *Cryptococcus neoformans* was found only in control insects (Figure 3A). *S. cerevisiae* was isolated from the gut content of *Vespa crabro* insects caught in Area 1 during the harvest 2020 sampling, and from *V. crabro* and *Vespula germanica* insects caught in Area 3 in the harvest 2021 sampling (Supplementary Table S3), and the frequency of isolation of this yeast species was positively correlated with the area surrounding the vineyard dedicated to woods including deciduous trees (Supplementary Figure S7). Further yeast species previously found in the vineyard (Morgan et al., 2017; Varela and Borneman, 2017), like *Lachancea thermotolerans* (9 isolates) and *M. pulcherrima* (653 isolates) were consistently found in both types of captured insects (IVC and IVW). Some species frequently found in association with insects (Meriggi et al., 2020) were well represented in wasps caught in both vineyards close and far from woods, such as species belonging to the *Starmerella*, *Meyerozyma*, *Pichia*, and *Wickerhamomyces* genera (Figure 3A). The frequency of identification of several yeast species was positively correlated with the percentage of the area surrounding the vineyard dedicated to wooded areas mostly composed of black locusts (*Candida maltosa* and *Zalaria obscura*), downy oaks (*C. parapsilosis/zeylanoides*), oaks (*Saccharomycopsis capsularis* and *Tetrapisispora blattae*), deciduous trees (*[Candida] zeylanoides*, *Candida* sp., *Pichia kudriavzevii*, *S. cerevisiae*), and poplars (*Hanseniaspora uvarum*, *M. pulcherrima*, *Metschnikowia* sp.; Supplementary Figure S7).

To assess the ecological potential of the isolated yeast species, we quantified the capability of strains representative of the isolated species to grow in various environmental settings they could face when residing in the wood, in anthropogenic areas, or in the wasp intestines (please refer to section 3.6 for further details). As a reference for the capability of strains to grow in the vineyard and cellar environment, we used three *S. cerevisiae* and one *Nakazawaea* sp. strains that were isolated previously from spontaneous fermentation of grapes harvested in Area 2 (identified as “wine” in Figure 3). The tested yeast isolates did not show significant differences among each other in their capabilities of growing in the various media (Wilcoxon–Mann–Whitney *p* > 0.05, Supplementary Figure S8). The comparison of different species revealed significant differences (Supplementary Table S5), which were particularly emphasized for a few species. *S. cerevisiae* was significantly less efficient in growing on arabinose and more efficient in growing on sucrose compared to other species. Contrarily, *S. ludwigii* was less performant in sucrose compared to other yeast species, whereas *Pichia fermentans/kluyveri* showed a reduced growth capability in YNB (with and without amino acids) compared to other species (Supplementary Table S5; Supplementary Figure S9). The PCA on growth capability data did not show a clear separation of yeast species according to the source of isolation (isolated only from insects caught from vineyards either close or far from wood or core mycobiota, permANOVA *p* > 0.05, Figure 3B). Most of the tested strains, also including the *S. cerevisiae* and *Nakazawaea* sp. strains isolated from wine must, showed intermediate growth capabilities, except for the strain representative of the *Rhodotorula mucilaginosa* species (number 51 in Figure 3B), which, according to the projection of the PCA variables on the first two coordinates of the PCA (Figure 3C) showed the highest capability of growing in any tested condition. The K-means analysis revealed 6 different clusters in the distribution of samples according to the PCA ordinates (Supplementary Figure S10A). The clusters were variously composed, including strains isolated from wine or the gut of wasps caught in vineyards close to wooded areas (yIVW), in vineyards used as controls (yIVC), or both (core; Supplementary Figure S10B). Cluster 6 encompassed only yIVW and core strains (*Aureobasidium pullulans*, *[Candida] zeylanoides*, *Candida* sp., *Metschnikowia* sp., *Nakazawaea wickerhamii*, *Pichia fermentans/kluyveri*, *Pichia kudriavzevii*, *Starmerella cellae*, *Starmerella* sp., *Wickerhamomyces anomalus*, and *Wickerhamomyces canadensis*), which were capable of growing in the presence of arabinose and sucrose, but performed poorly in minimal media (Wilcoxon–Mann–Whitney results are reported in Supplementary Table S6; Supplementary Figure S10B). Conversely, cluster 5, encompassing the wine isolates and strains belonging to the yIVW, yIVC, and core groups, performed better in minimal media but showed limited growth in the presence of arabinose and sucrose (Supplementary Table S6; Supplementary Figure S10B).

## Discussion

The presence of woods in the proximity of vineyards influences multiple aspects of yeast populations vectored by social insects. Woodlands potentially provide the wasps with a higher amount and more variegate substrates than anthropogenic areas, such as flowers, soil, fruit, small animals, and decaying material, which are a source of natural yeasts (Mozzachiodi et al., 2022). Potentially as a consequence of the broader range of sources, we observed that insects caught in vineyards close to wooded areas bear more abundant and more diverse yeast populations than insects caught in vineyards distant from wooded areas (Figure 2A). The higher abundance of yeast cells could be ascribed to either the fact that wasps (i) feed on substrates rich in yeasts and food ingested by wasps promotes the growth of yeast cells in the insect’s intestine or (ii) by feeding on multiple substrates provided by forest habitat, collect the yeast cells present on every substrate, hence enriching their mycobiota. The latter hypothesis would also provide a justification for the higher yeast biodiversity observed in wasps caught in vineyards close to woods. Also, the comparison of the yeast population compositions showed a clear differentiation of insects caught in vineyards close and far from woodlands (Figure 3A).

The other factors which are already known to influence yeast populations in the vineyard, namely the vintage and the geographical area (Capozzi et al., 2015; Jara et al., 2016; Chalvantzi et al., 2021), were confirmed to be correlated with the number of yeast species and the composition of yeast populations. The differences between yeast abundances in insects caught in vineyards close and far from woods were observed when comparing insects of different geographical areas, but not when comparing insects grouped according to the vintage. Unexpectedly, the same differences between the abundance, diversity, and composition of yeast communities associated with geographically distant vineyards were observed also between the insects caught in the vineyards of the same province, only 10 km apart. Even more strikingly, the yeast abundances differed only between the two couples of vineyards located in the same province and not with the vineyard located more than 75 km apart (Area 2). This observation seemingly contradicts previous studies highlighting the association between yeast populations and geographical location (Bokulich et al., 2014). Indeed, our results suggest that the presence of wooded areas, rather than the distance between vineyards, plays a vital role in defining yeast populations. The currently available information on the land cover, topographical, and pedological characteristics of the studied vineyards, allowed us to highlight some of the environmental factors that could be associated with the definition of the insect and yeast biodiversity found in vineyards. However, especially considering that some of these factors seem to have opposite associations with the insect and yeast biodiversities (e.g., the presence of woods mostly composed of black locusts, negatively correlated with the insect biodiversity and positively with the frequency of isolation of some yeast species), it will be necessary to extend the sampling to other vineyards and gather in-depth information on the environmental matrix in order to draw appropriate conclusions.

The proximity of the vineyard to wooded areas influences not only the abundance and diversity of yeast populations but also promotes the vectoring of yeast species known to have a positive role in the alcoholic fermentation of the must. The species mostly responsible for alcoholic fermentation, *Saccharomyces cerevisiae*, has been isolated only from insects caught in vineyards close to woods (Figure 3A). On the other hand, yeasts found only in insects caught in vineyards far from woodlands belong to species detrimental to the fermentation of the must, such as *Zygoascus hellenicus* (Barata et al., 2012), or even of human health, such as *Cryptococcus neoformans* (Figure 3A; Kronstad et al., 2011).

Overall, social wasps confirm to be a crucial vector for yeast species playing relevant roles in the ecology of yeasts and the wine-making process, such as some species consistently present in the gut of analyzed insects, independently of the type of the source vineyards. For instance, the *M. pulcherrima* species, represented by 63 isolates (Supplementary Table S3; 13% of the strains isolated in this study), plays an important role in wine-making by expressing several enzymes responsible for amino acids degradation, hence promoting the release of nitrogen necessary for *Saccharomyces cerevisiae* growth (Tufariello et al., 2021). *Metschnikowia pulcherrima* also influences the organoleptic characteristics of the final product by modulating the release of terpenes (Morata et al., 2019) and volatile thiols (Zott et al., 2011). Another species consistently found in insects’ guts, *Hanseniaspora uvarum*, can influence the complexity and richness of yeast populations by competing with other fungi such as *Botrytis cinerea* and can confer fruity aromas and other organoleptic characteristics to the wine (Guaragnella et al., 2020). An additional example of yeast species of oenological interest found in wasp intestines is *Lachancea thermotolerans*: strains of this species can produce lactic acid and ethanol through alcoholic fermentation, hence showing great potential for the production of less acidic wines (Vicente et al., 2021).

The performed phenotypic analysis allowed us to confirm that the wasp intestine bear yeast species characterized by a variegate range of traits (Dapporto et al., 2016). Furthermore, our findings revealed the existence of a group of wasp gut isolates capable of growing on plant substrates (hemicellulose and pectin, rich in arabinose, and plant roots, fruits, and nectars, rich in sucrose) but with a limited ability to grow in minimal media. Interestingly, this group of yeast species did not include strains distinctive of insects caught in vineyards used as controls. Overall, this result supports the hypothesis that woods provide some nutrients (sucrose and arabinose) that are not present or limited in other environments, thus allowing the survival of yeast strains and species otherwise not capable of surviving in anthropogenic environments. This preliminary information is promisingly relevant for the disclosure of yeast ecology, and future research should be aimed at better characterizing the characteristics of the environments surrounding the vineyards. Further studies are required to assess whether specific factors associated with the presence of wooded areas (e.g., plant species, age of the forest) are responsible for the differences observed among yeast populations vectored by insects. In particular, it will be necessary to determine which elements of the environmental matrix are associated with the vector and yeast biodiversity present in the vineyard and the cellar. In this optic, delving into the differences in the yeast populations vectored by different social wasp species holds the promise of easing the identification of the factors of the environmental matrix influencing the composition of yeast populations. Aiming at this, multi-disciplinary approaches will be fundamental to consider the ecology and feeding habits of the different insect species, the pedological along with the land cover characteristics of the environment, and the ecological potential of yeast species.

## Data availability statement

The original contributions presented in the study are included in the article/Supplementary material, further inquiries can be directed to the corresponding authors.

## Author contributions

FB, AL, and IS: conceptualization. BV, FB, LC, and IS: data collection, data analysis, and formal analysis. BV and IS writing—original draft. FB, LC, AL, IS, and BV: writing—review and editing. BV and IS: visualization. All authors contributed to the article and approved the submitted version.

## Funding

The study was funded by Fondazione CRT-Cassa di Risparmio di Torino. BV was supported by a fellowship granted by the PON “Ricerca e Innovazione” (National Operative Programme Research and Innovation) 2014–2020, from the Italian Ministry of University and Research (MUR).

## Conflict of interest

The authors declare that the research was conducted in the absence of any commercial or financial relationships that could be construed as a potential conflict of interest.

## Publisher’s note

All claims expressed in this article are solely those of the authors and do not necessarily represent those of their affiliated organizations, or those of the publisher, the editors and the reviewers. Any product that may be evaluated in this article, or claim that may be made by its manufacturer, is not guaranteed or endorsed by the publisher.

## Footnotes

^1^ www.geoportale.piemonte.it

^2^ http://www.ncbi.nlm.nih.gov/
