# Supplementary material for: Forests influence yeast populations vectored by insects into vineyards
Source: Front Microbiol. 2022 Nov 21;13:1039939. doi: 10.3389/fmicb.2022.1039939 (PMC9719976; doi:10.3389/fmicb.2022.1039939)
Supplement: Supplementary file 5 [file Data_Sheet_1.pdf]

Supplementary Material

Forests influence yeast populations vectored by insects into vineyards

Beatrice Valentini, Luca Pietro Casacci, Anna Luganini, Francesca Barbero, Irene Stefanini

Department of Life Sciences and Systems Biology, University of Turin, Turin, Italy

This pdf file includes:

Supplementary Figures

Supplementary figure 1: Sampled areas.
Supplementary figure 2: Comparison of yeast abundances observed in insects' guts.
Supplementary figure 3: Distribution of insects resulting in yeast isolation.
Supplementary figure 4: Alpha diversities of yeast populations in insects' guts.
Supplementary figure 5: PCoA based on Jaccard distances among yeast populations in sampled insects.
Supplementary figure 6: PCoA based on Unifrac distances among yeast populations in sampled insects.
Supplementary figure 7: Correlations between the frequency of isolation of yeast species and land cover, topographic, and pedological characteristics of the vineyards and their surrounding areas.
Supplementary figure 8: Growth capabilities of yeast species isolated from the intestine of the analyzed insects.
Supplementary figure 9: Comparison of the growth ratio of strains grouped according to the species.
Supplementary figure 10: Yeast species clustering according to the quantified traits.

Supplementary Tables

Supplementary table 1: Details on the vineyards sampled in this study (provided as a separate file).
Supplementary table 2: Details on the insects caught in this study (provided as a separate file).
Supplementary table 3: Details on the yeast strains isolated in this study (provided as a separate file).
Supplementary table 4: Strains used as the representatives of the species for phenotypic tests.
Supplementary table 5: Comparison of yeasts' phenotypic characteristics associated with different capabilities of growing in different media (provided as a separate file).
Supplementary table 6: Results of Wilcoxon-Mann-Whitney tests on phenotypes of tested strains.

Supplementary results

Supplementary statistical results: Detailed results of the statistical analyses carried out in the study.

Supplementary Figures and Tables

Supplementary Figure 1. Sampled areas. **A)** Red and yellow indexes indicate vineyards close to woods ("VW", Vineyards close to Woods) or far from woods ("ctrl", "VC", Vineyards used as the Control), respectively. The map was obtained with Google Earth Version 9.168.0.0 (April 2020).

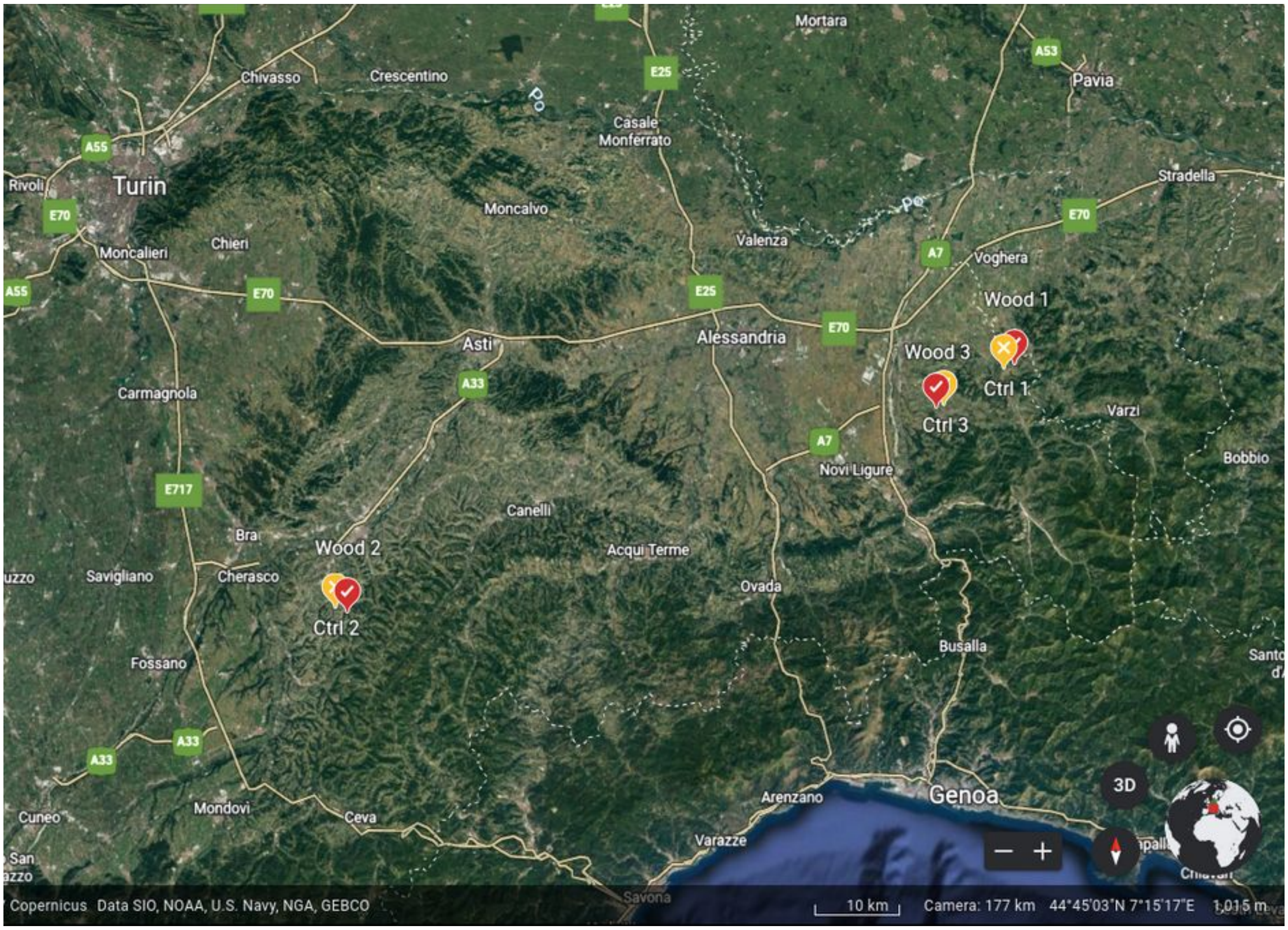

Supplementary Figure 1. Sampled areas. **B)** Land cover (percentages) of the area surrounding (within a 500mt radium) the vineyards under investigation and **C)** boxplots comparing the vineyards grouped according to the geographical area. No significant differences were found between the variables in the compared vineyards (Wilcoxon-Mann-Whitney test, fdr>0.05). VC= Vineyards used as the Control; VW= Vineyards close to Woods. “dec. trees”= deciduous trees.

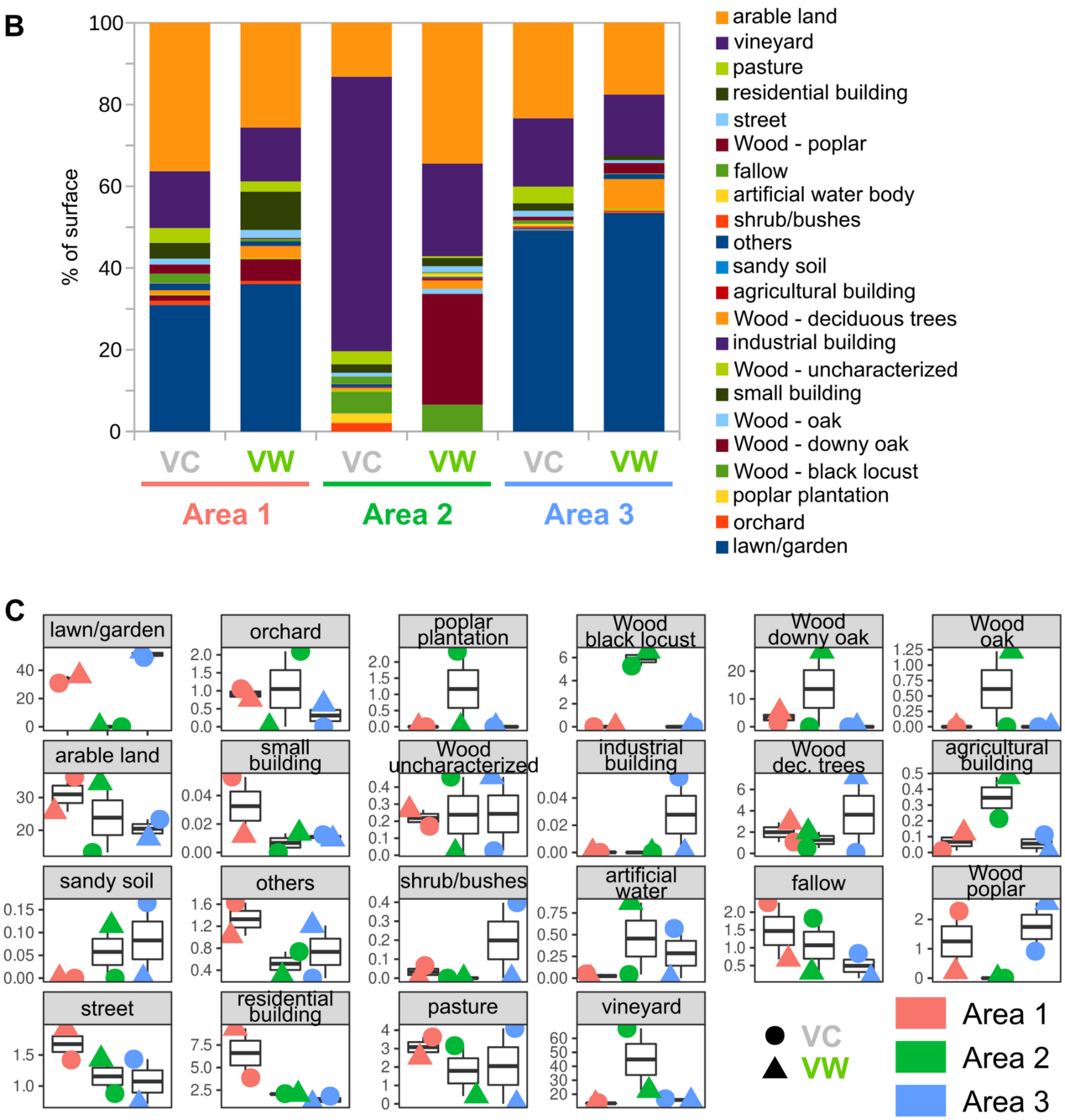

Supplementary Figure 1. Sampled areas. **D)** Pedological and topological characteristics of the vineyards under investigation and **E)** boxplots comparing the vineyards grouped according to the geographical area. No significant differences were found between the variables in the compared vineyards (Wilcoxon-Mann-Whitney test, fdr>0.05). VC= Vineyards used as the Control; VW= Vineyards close to Woods.

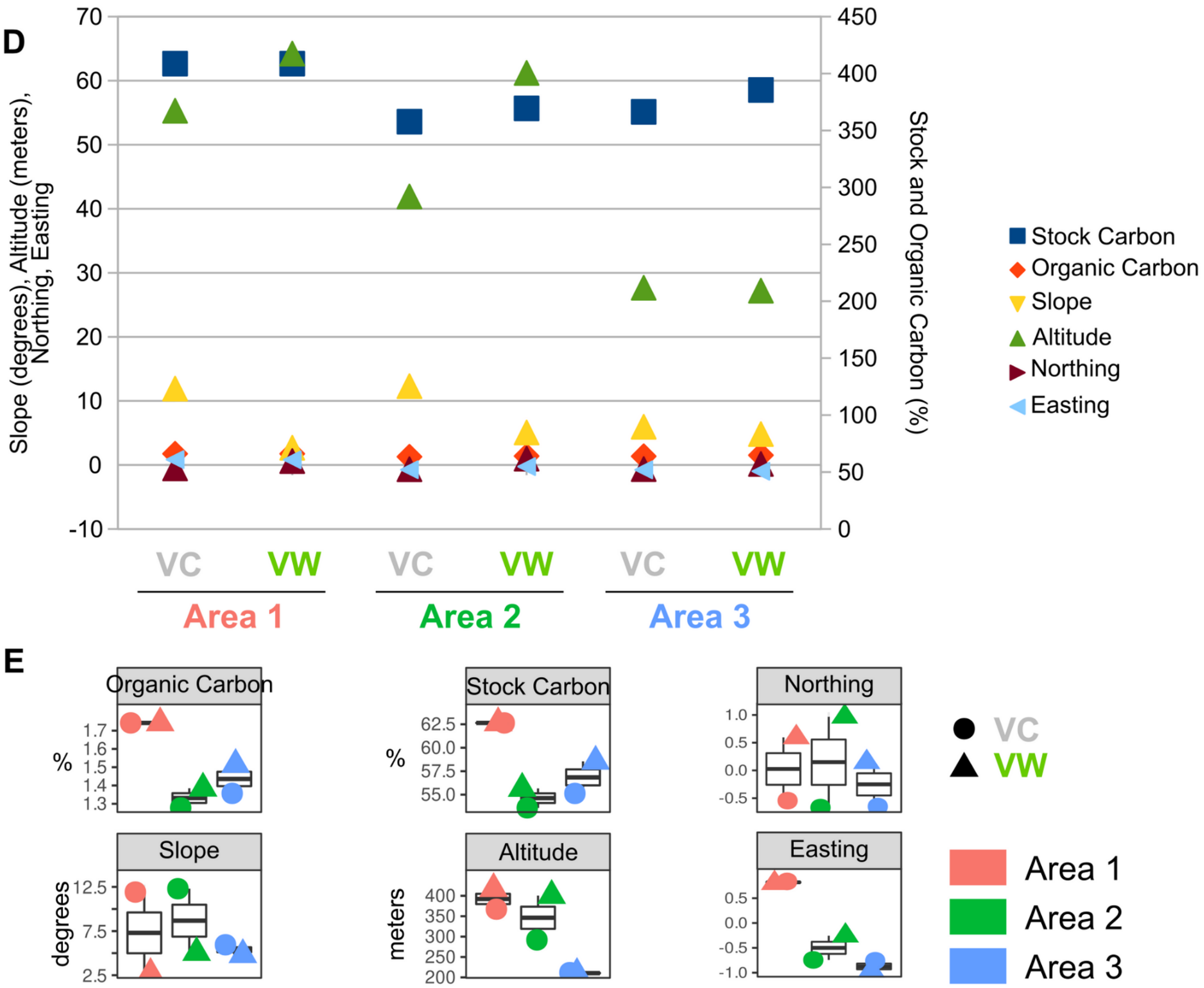

Supplementary Figure 2. Comparison of yeast abundances observed in insects' guts. The number CFUs (Colony Forming Units) per insect was compared between insects grouped according to **A)** the area of sampling, **B)** the year of sampling; **C)** the species of the insect; and **D)** the season of sampling. *Wilcoxon-Mann-Whitney fdr<0.05.

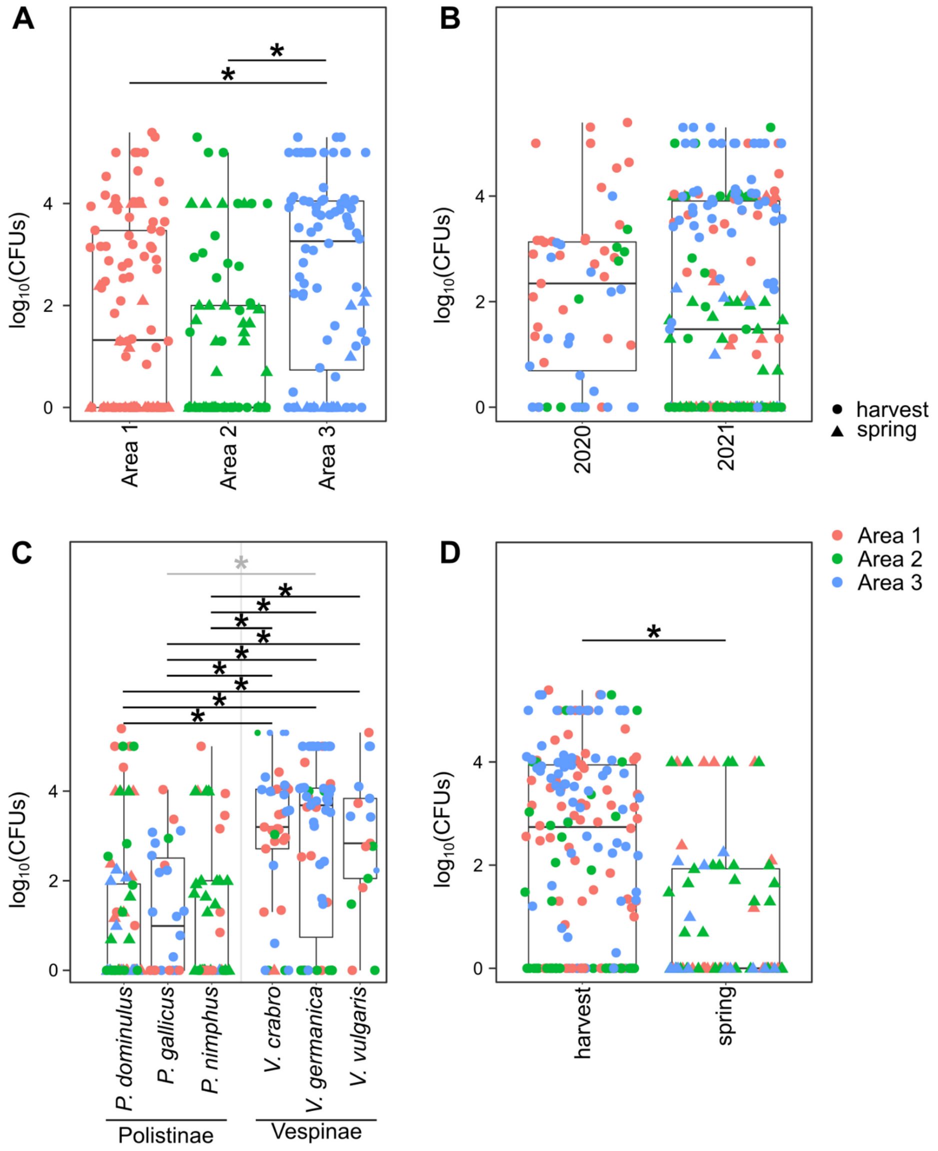

Supplementary Figure 3. Distribution of insects resulting in yeast isolation. For each group indicated in the y-axis, the black bar indicates the percentage of insects providing yeast isolates, the gray bar indicates the percentage of insects not providing yeast isolates. The numbers written on the bars correspond to the number of insects. IVC= Insects caught in Vineyards used as Controls; IVW= Insects caught in Vineyards close to Woods.

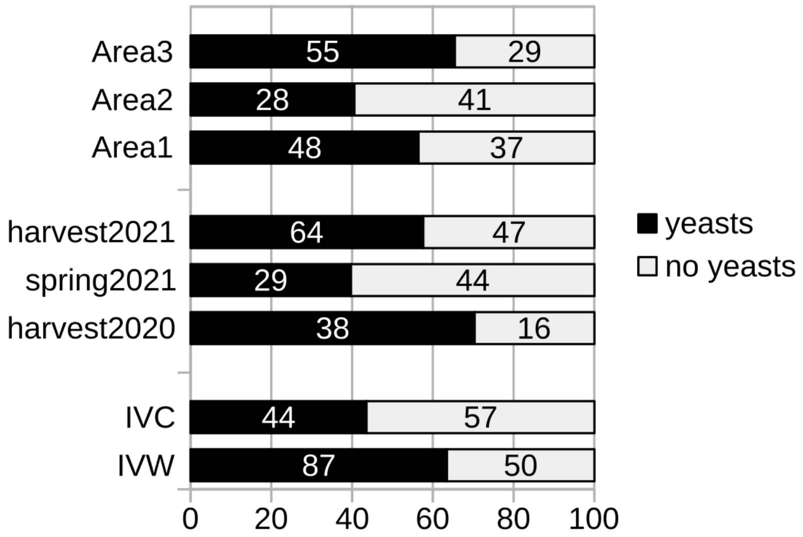

Supplementary Figure 4. Alpha diversities of yeast populations in insects' guts. A) Comparison of the number of yeast species found in insects grouped according to the collection area. **B)** Comparison of the number of yeast species found in insects grouped according to the time of collection. **C)** Comparison of the number of yeast species found in insects grouped according to the insect species. * Wilcoxon-Mann-Whitney fdr<0.05. IVC= Insects caught in Vineyards used as Controls; IVW= Insects caught in Vineyards close to Woods.

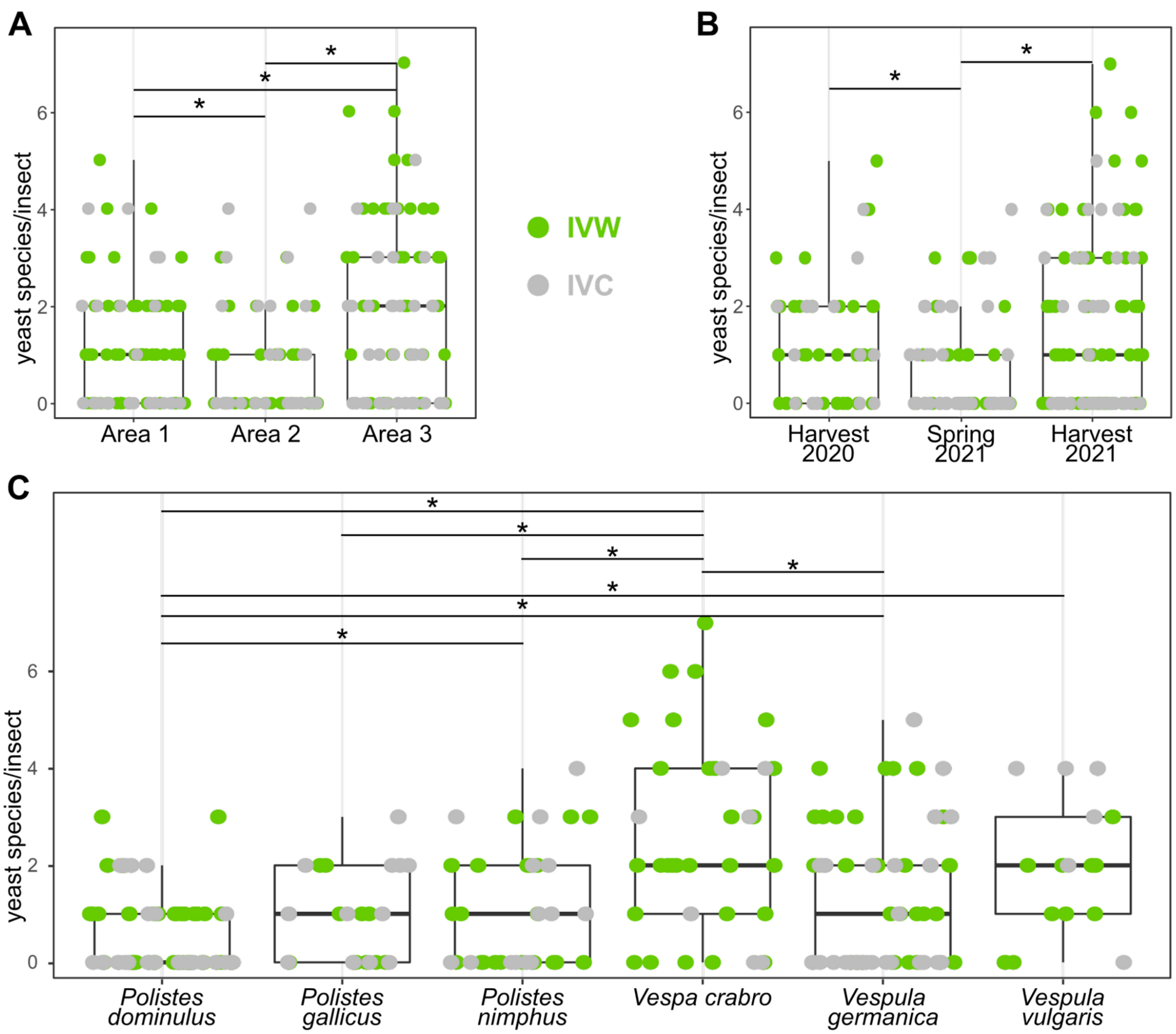

Supplementary Figure 5. PCoA based on Jaccard distances among yeast populations in sampled insects. First two Coordinates of the PCoA on the Jaccard distances among yeast populations of insects' gut, with samples colored according to **A)** the sampling area, **B)** the year of sampling, and **C)** the insect species.

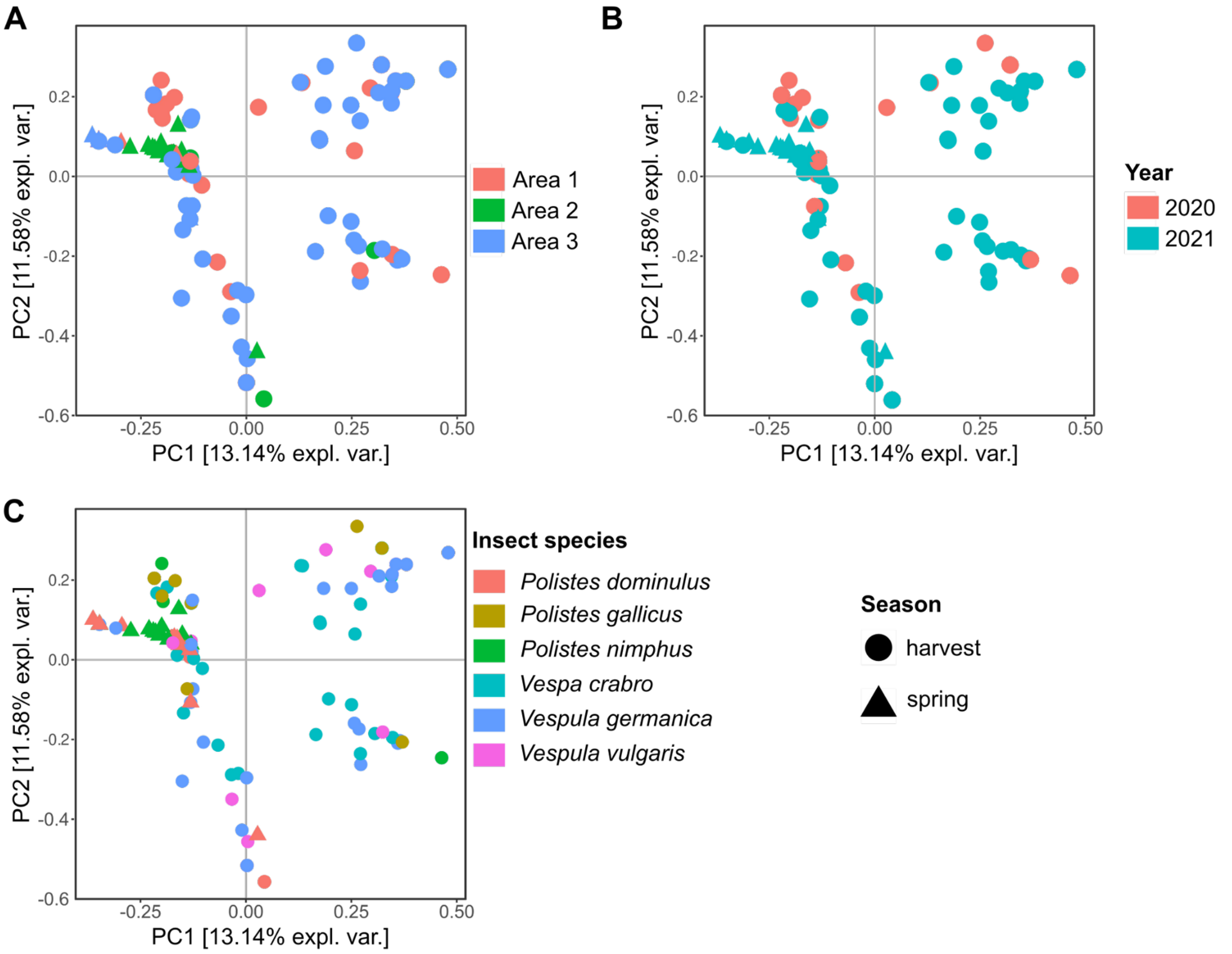

<u>Supplementary Figure 6</u>. PCoA based on Unifrac distances among yeast populations in sampled insects. First two Coordinates of the PCoA on the Unifrac distances among yeast populations of insects' gut, with samples colored according to **A)** wood or control insects, **B)** the sampling area, **C)** the year of sampling, and **D)** the insect species. IVC= Insects caught in Vineyards used as Controls; IVW= Insects caught in Vineyards close to Woods.

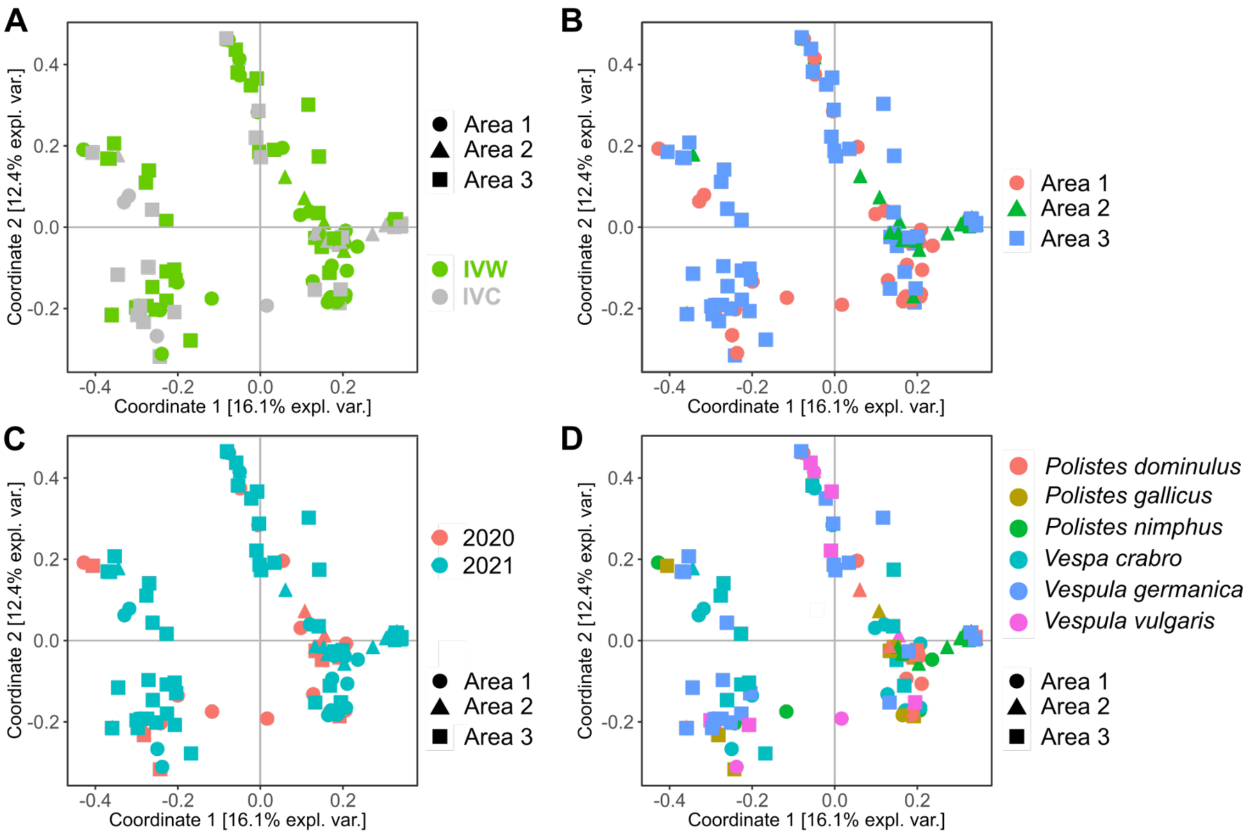

Supplementary Figure 7. **Correlations between the frequency of isolation of yeast species and land cover, topographic, and pedological characteristics of the vineyards and their surrounding areas.** Correlations were assessed by means of Spearman correlation analysis between the frequency of isolation of the yeast species (expressed as the number of insects bearing the yeast species on the total number of analyzed insects) and the land cover, topographic, and pedological information on the vineyards and the area surrounding them (within a 500m radius). Only significant correlations (fdr<0.05) are shown.

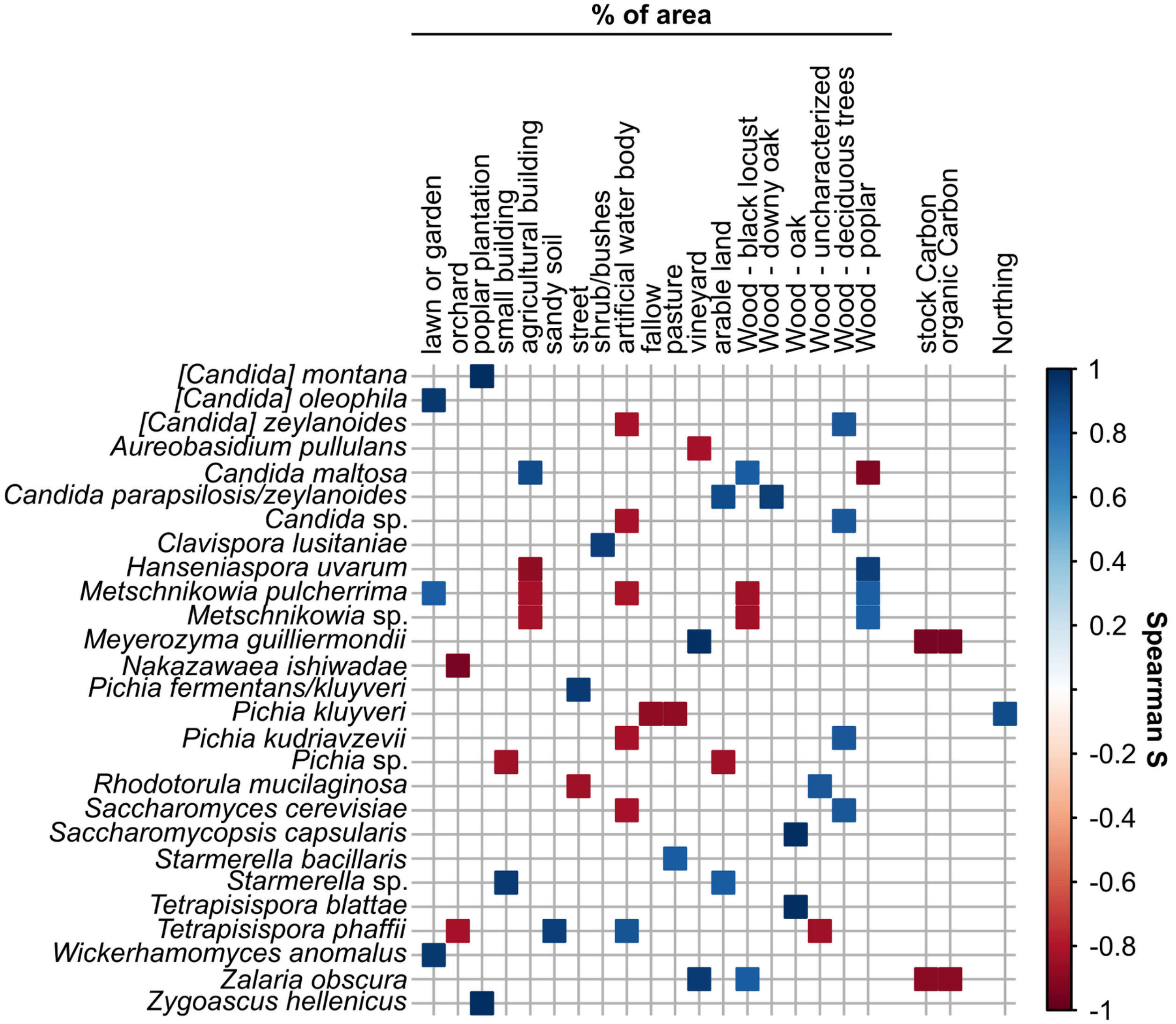

Supplementary Table 4. Strains used as the representatives of the species for phenotypic tests. At least one strain for each species isolated in this study was selected as the representative of the species. In the table are reported: the ID of the selected strain (corresponding to the ID reported in **Supplementary Table 3**), the species, the GenBank Accession number of the ITS1 sequence, the group (yIVW- or yIVC- specific, or "core", as indicated in **Fig. 3**), and the "Species ID" of the strain indicated in the plots reporting the data on growth capabilities (**Fig. 3B**, Supplementary Figure 8), and the Cluster number (see Supplementary Figure 10) [a]Square brackets ([ ]) around a genus indicate that the name awaits appropriate action by the research community to be transferred to another genus. yIVC= yeasts belonging to species isolated only from Insects caught in Vineyards used as Controls; yIVW= yeasts belonging to species isolated only from Insects caught in Vineyards close to Woods; core= yeasts belonging to species isolated both from IVW and IVC; wine= strains used as the reference, previously isolated from spontaneous fermentation of grape musts of Area 2.

Strain_ID	Species	GenBank Access	Group	Species ID	Cluster
C_IC_3.2	*Clavispora lusitaniae*	OP663282	yIVC	1	5
C_IC_4.2	*Clavispora lusitaniae*		yIVC	2	5
C_TH21_6.4	*Clavispora lusitaniae*		yIVC	3	1
C_TH21_12.1	*Cryptococcus neoformans*	OP663221	yIVC	4	1
C_IC_8.3	*Pichia bruneiensis*	OP661215	yIVC	5	5
C_IC_8.2	*Saturnispora diversa*		yIVC	6	4
B_TH21_25.1	*[Candida]*[a] *oleophila*	OP663214	core	7	1
B_ICS21_15.2	*Aureobasidium pullulans*	OP663268	core	8	4
B_ICS21_15.4	*Aureobasidium pullulans*	OP663246	core	9	4
B_ICS21_15.5	*Aureobasidium pullulans*	OP663216	core	10	4
B_ICS21_15.6	*Aureobasidium pullulans*	OP663217	core	11	4

B_TH21_24.4	*Aureobasidium pullulans*	OP663215	core	12	6
C_TS21_9.1	*Aureobasidium pullulans*		core	13	6
B_RS21_12.1	*Candida maltosa*		core	14	4
B_TH21_19.6	*Candida tropicalis*	OP663220	core	15	1
B_RH21_12.1	*Hanseniaspora uvarum*	OP663224	core	16	5
B_TH21_10.4	*Hanseniaspora uvarum*	OP663225	core	17	1
B_TH21_12.2	*Hanseniaspora uvarum*	OP663223	core	18	5
B_TH21_12.7	*Hanseniaspora uvarum*	OP663241	core	19	5
B_TH21_19.2	*Hanseniaspora uvarum*	OP663222	core	20	2
C_TH21_3.1	*Hanseniaspora uvarum*	OP663249	core	21	2
C_GH21_1.3	*Lachancea thermotolerans*	OP663227	core	22	1
B_IC_9.1	*Metschnikowia pulcherrima*	OP663251	core	23	4
B_TH21_2.4	*Metschnikowia pulcherrima*	OP663253	core	24	1
B_IC_19.4	*Metschnikowia sp*	OP663247	core	25	1
B_ICH21_20.1	*Metschnikowia sp*	OP663265	core	26	1
B_TH21_18.1	*Metschnikowia sp*	OP663266	core	27	1
B_TH21_21.1	*Metschnikowia sp*	OP663260	core	28	1
B_TH21_3.3	*Metschnikowia sp*	OP663286	core	29	1
B_TH21_6.7	*Metschnikowia sp*	OP663257	core	30	6
C_GS21_15.1	*Metschnikowia sp*	OP650086	core	31	1
C_IC_5.1	*Metschnikowia sp*	OP663283	core	32	5
C_ICH21_1.2	*Metschnikowia sp*	OP663287	core	33	5
C_ICH21_2.4	*Metschnikowia sp*	OP663274	core	34	6
C_T_7.1	*Metschnikowia sp*	OP663279	core	35	5
C_TH21_10.2	*Metschnikowia sp*	OP663258	core	36	1

C_TH21_4.6	*Metschnikowia sp*	OP663288	core	37	1
C_TH21_8.3	*Metschnikowia sp*	OP663259	core	38	6
C_TH21_9.2	*Metschnikowia sp*	OP663269	core	39	1
C_TH21_13.3	*Meyerozyma guilliermondii*	OP663228	core	40	2
C_TH21_7.1	*Nakazawaea ishiwadae*	OP663261	core	41	5
B_IC_13.2	*Pichia fermentans/kluyveri*	OP663235	core	42	6
B_IC_14.1	*Pichia fermentans/kluyveri*	OP663264	core	43	6
B_IC_16.1	*Pichia fermentans/kluyveri*	OP663271	core	44	4
B_IC_17.2	*Pichia fermentans/kluyveri*	OP663263	core	45	6
C_T_5.1	*Pichia fermentans/kluyveri*	OP663280	core	46	6
B_TH21_20.3	*Pichia sp*	OP663273	core	47	4
B_TH21_7.6	*Pichia sp*	OP663267	core	48	1
B_TH21_8.5	*Pichia sp*		core	49	1
C_GH21_1.6	*Pichia sp*	OP663238	core	50	1
B_TH21_21.2	*Rhodotorula mucilaginosa*	OP663239	core	51	3
B_IC_13.4	*Starmerella bacillaris*	OP663275	core	52	5
B_IC_14.2	*Starmerella bacillaris*	OP663276	core	53	5
B_IC_4.5	*Starmerella bacillaris*	OP663256	core	54	5
B_IC_8.1	*Starmerella bacillaris*	OP663281	core	55	5
B_IC_9.2	*Starmerella bacillaris*	OP663277	core	56	5
B_ICH21_3.2	*Starmerella bacillaris*	OP663232	core	57	1
C_IC_5.2	*Starmerella bacillaris*	OP663284	core	58	5
C_IC_6.1	*Starmerella bacillaris*	OP663278	core	59	5
B_RH21_8.4	*Starmerella cellae*	OP663213	core	60	6
B_ICH21_1.2	*Starmerella sp*	OP663272	core	61	5
B_R_8.4	*Starmerella sp*	OP650085	core	62	5

B_TH21_10.8	*Starmerella sp*	OP650088	core	63	6
C_ICH21_1.3	*Starmerella sp*		core	64	6
C_ICH21_3.2	*Starmerella sp*	OP663248	core	65	6
C_TH21_10.5	*Starmerella sp*		core	66	6
C_TH21_13.2	*Starmerella sp*		core	67	5
C_TH21_15.2	*Starmerella sp*		core	68	1
B_IC_6.4	*Wickerhamomyces anomalus*		core	69	6
B_TH21_17.1	*Wickerhamomyces anomalus*	OP663230	core	70	1
B_TH21_5.1	*Wickerhamomyces anomalus*	OP663231	core	71	1
B_RS21_2.1	*Zalaria obscura*	OP663255	core	72	4
C_GS21_15.2	*Zalaria obscura*	OP650087	core	73	1
BARBM16.3	*Nakazawaea sp*	OP663289	wine	74	5
BARBM_16.1	*Saccharomyces cerevisiae*	OP663244	wine	75	5
BARBM_16.4	*Saccharomyces cerevisiae*	OP663245	wine	76	5
BARBM16.2	*Saccharomyces cerevisiae*	OP663242	wine	77	5
B_TH21_2.3	*[Candida]*[a] *membranifaciens*	OP663212	yIVW	78	1
B_IC_7.3	*[Candida]*[a] *zeylanoides*		yIVW	79	6
B_T_9.3	*[Candida]*[a] *zeylanoides*		yIVW	80	4
B_IC_6.5	*Ambrosiozyma angophorae*		yIVW	81	5
B_TH21_7.4	*Candida norvegica*	OP663218	yIVW	82	2
B_ICH21_2.2	*Candida sp*		yIVW	83	6
B_TH21_1.2	*Candida sp*	OP663219	yIVW	84	6
B_TH21_12.4	*Candida sp*		yIVW	85	5
B_TH21_21.3	*Candida sp*		yIVW	86	6
B_TH21_28.1	*Candida sp*		yIVW	87	5
B_TH21_29.1	*Candida sp*	OP663285	yIVW	88	6

B_TH21_3.2	*Candida sp*		yIVW	89	1
B_TH21_4.4	*Candida sp*		yIVW	90	6
B_TH21_6.4	*Candida sp*	OP663250	yIVW	91	6
B_IC_8.2	*Hanseniaspora valbyensis*		yIVW	92	5
B_TH21_11.4	*Lachancea kluyveri*	OP663226	yIVW	93	5
B_T_6.1	*Nakazawaea wickerhamii*	OP663262	yIVW	94	6
B_TH21_4.3	*Pichia kluyveri*	OP663236	yIVW	95	1
B_IC_9.6	*Pichia kudriavzevii*	OP663252	yIVW	96	6
B_TH21_8.2	*Pichia kudriavzevii*	OP663237	yIVW	97	5
B_IC_6.2	*Saccharomyces cerevisiae*	OP663243	yIVW	98	5
B_TH21_11.2	*Saccharomyces cerevisiae*	OP663240	yIVW	99	5
B_TH21_12.1	*Saccharomyces cerevisiae*	OP663233	yIVW	100	1
B_TH21_24.1	*Saccharomyces cerevisiae*	OP663234	yIVW	101	5
B_IC_2.3	*Saccharomycodes ludwigii*		yIVW	102	5
B_IC_9.3	*Saccharomycodes ludwigii*	OP663270	yIVW	103	5
B_TH21_15.1	*Wickerhamomyces canadensis*	OP663229	yIVW	104	6
B_TH21_5.2	*Wickerhamomyces sp*		yIVW	105	2
B_TH21_9.3	*Wickerhamomyces sp*	OP663254	yIVW	106	1
B_IC_4.4	*Zygosaccharomyces bailii*		yIVW	107	5

Supplementary Figure 8. Growth capabilities of yeast species isolated from the intestine of the analyzed insects. The growth capabilities of the strains, selected as representative of every yeast species isolated in this study, were tested in three independent biological replicates. **A-B)** growth rates in the rich medium YP (1% Yeast extract, 2% Peptone) supplemented with 2% arabinose or **C-D)** supplemented with 2% sucrose; growth rates in the minimum medium YNB (Yeast Nitrogen Base) without **(E-F)** and with **(G-H)** the supplementation of amino acids. The Optical Density (OD) of the strain culture was measured at 24h and 48h after the inoculum. The plots report the average ratio calculated as the OD of the yeast culture in the tested medium divided by the OD of the yeast culture in YPD (YP supplemented with 2% glucose). Error bars indicate the standard error. Three *Saccharomyces cerevisiae* and one *Nakazawaea* sp. strains isolated from spontaneous fermentations carried out on the musts from the vineyard of Area 2 were used as references. To improve the clarity of the plot, the strains are indicated with the ID reported in Supplementary Table 4, rather than with the isolate ID or the species name. Bars are colored according to the group of strains/species as indicated in Supplementary Table 4 and **Figure 3**.

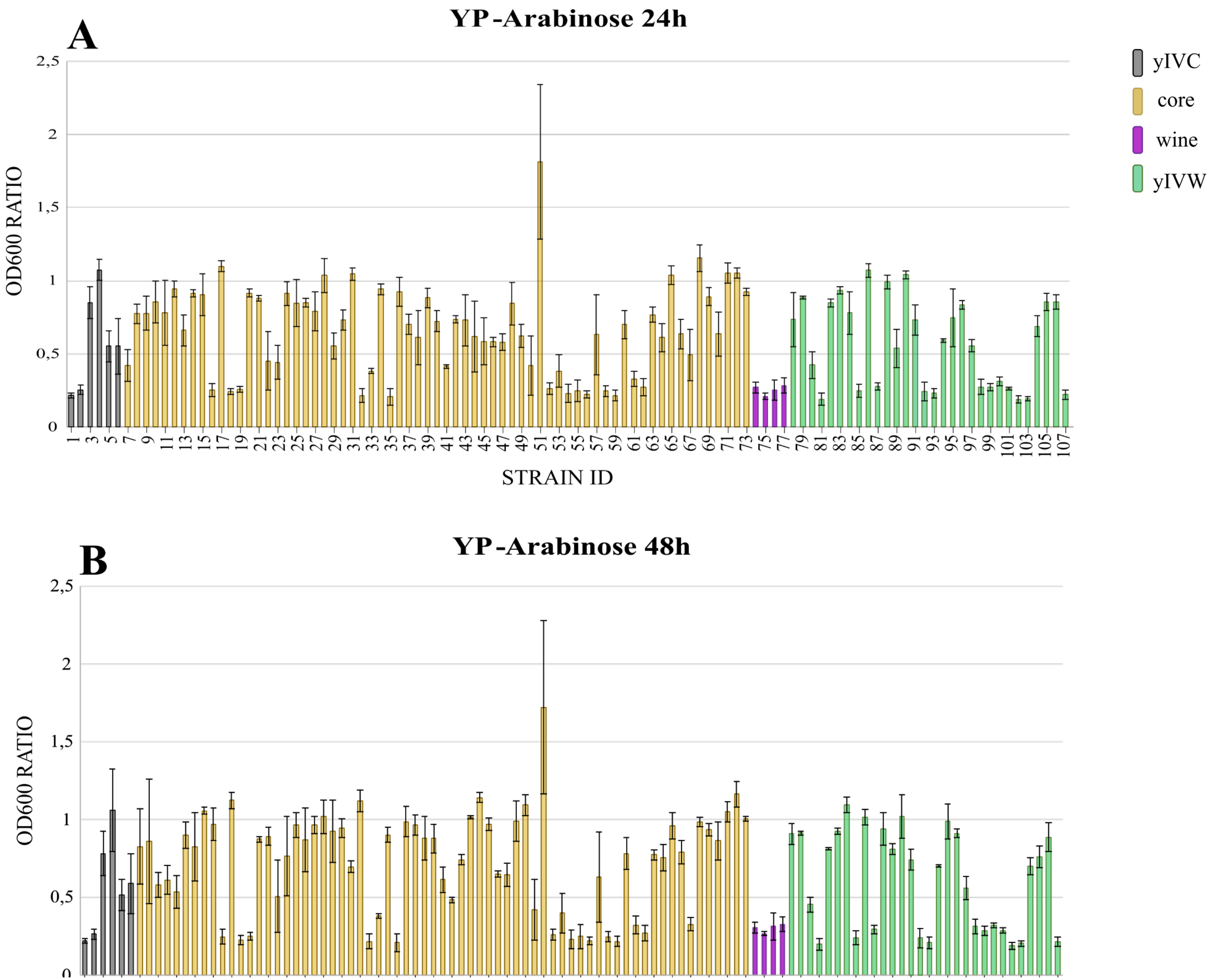
A
YP-Arabinose 24h
OD600 RATIO
0
0,5
1
1,5
2
2,5
STRAIN ID
yIVC
core
wine
yIVW
B
YP-Arabinose 48h
OD600 RATIO
0
0,5
1
1,5
2
2,5

C

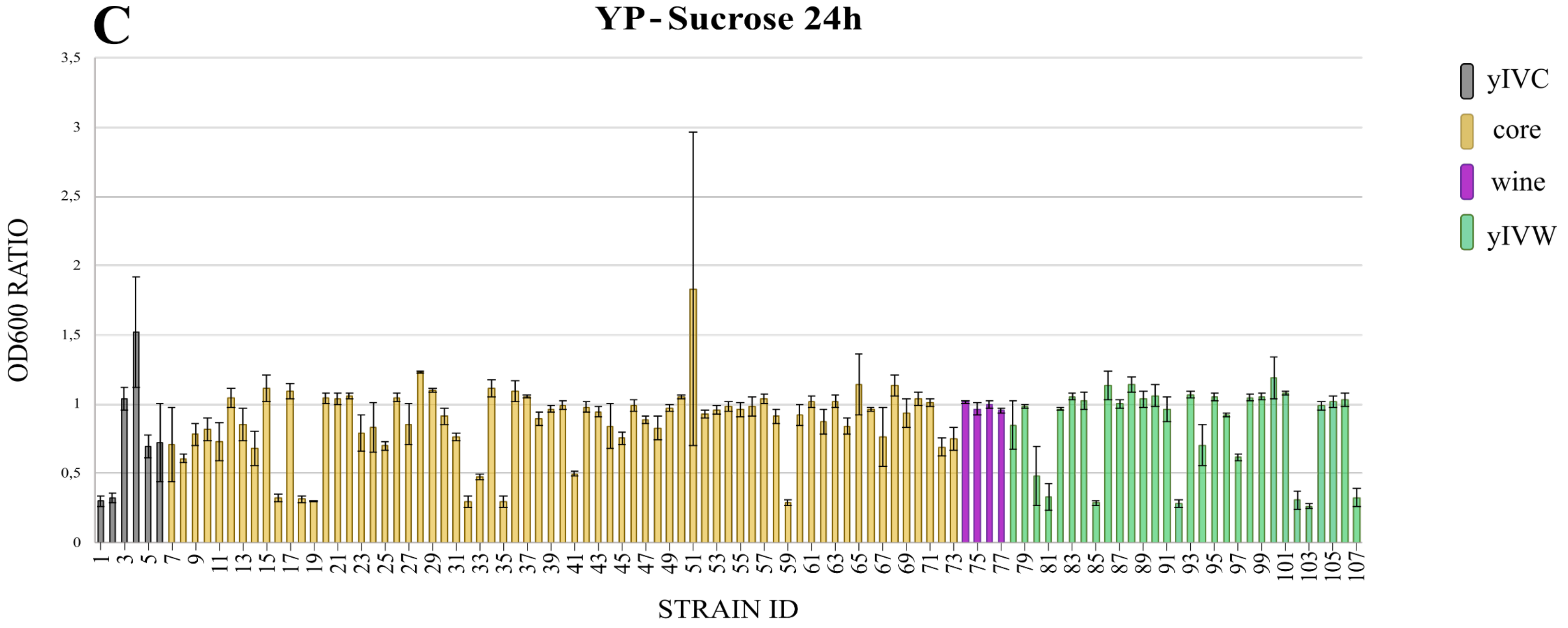
YP - Sucrose 24h
OD600 RATIO
STRAIN ID
yIVC
core
wine
yIVW

D

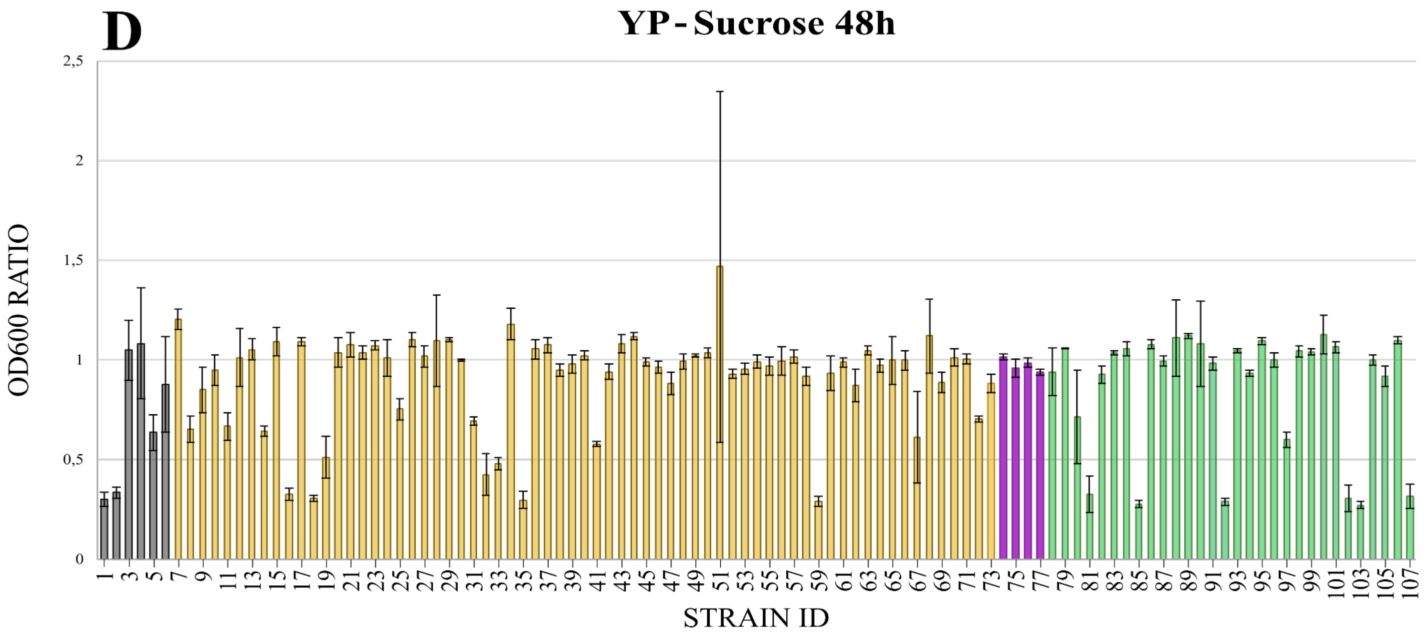
YP - Sucrose 48h
OD600 RATIO
STRAIN ID

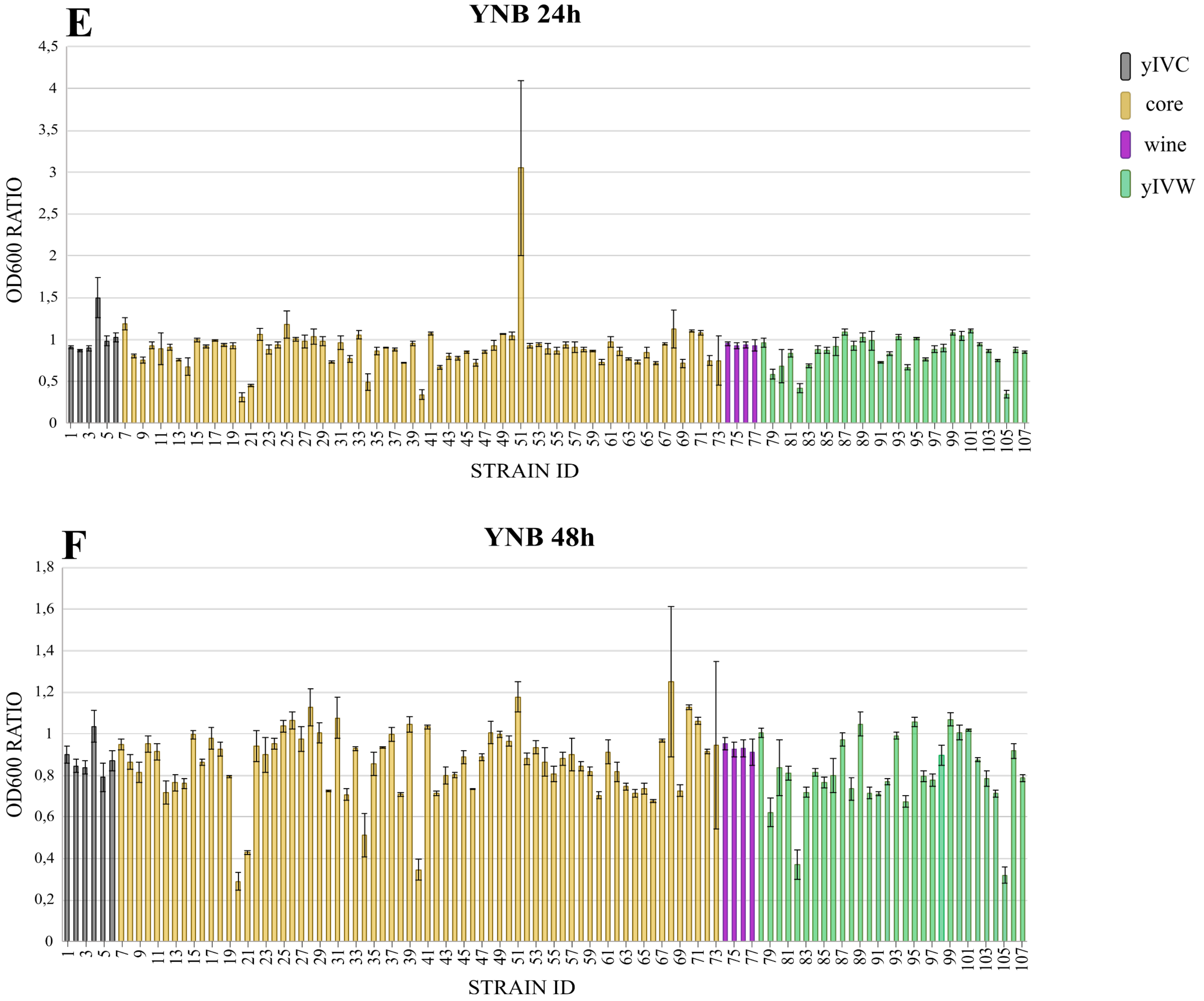
E
YNB 24h
OD600 RATIO
STRAIN ID
yIVC
core
wine
yIVW
F
YNB 48h
OD600 RATIO
STRAIN ID

G

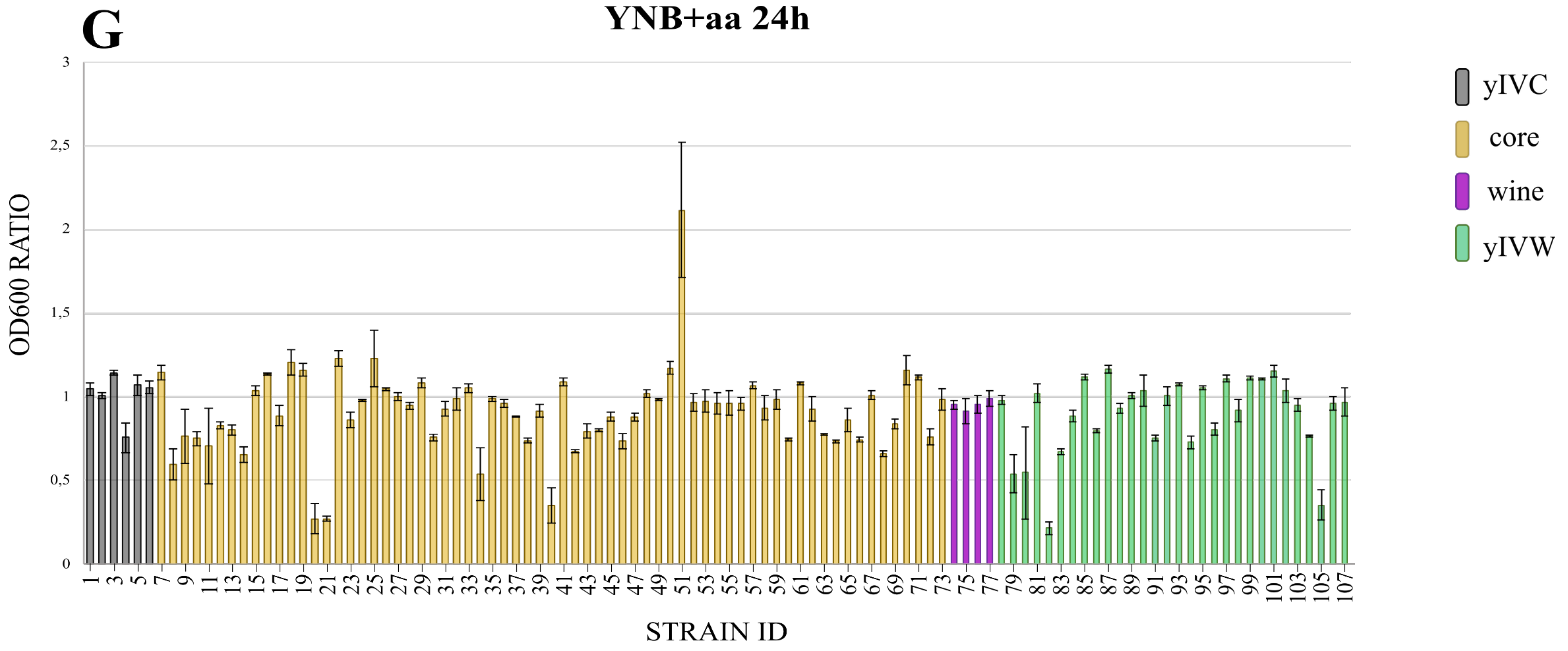
YNB+aa 24h
OD600 RATIO
STRAIN ID
yIVC
core
wine
yIVW

H

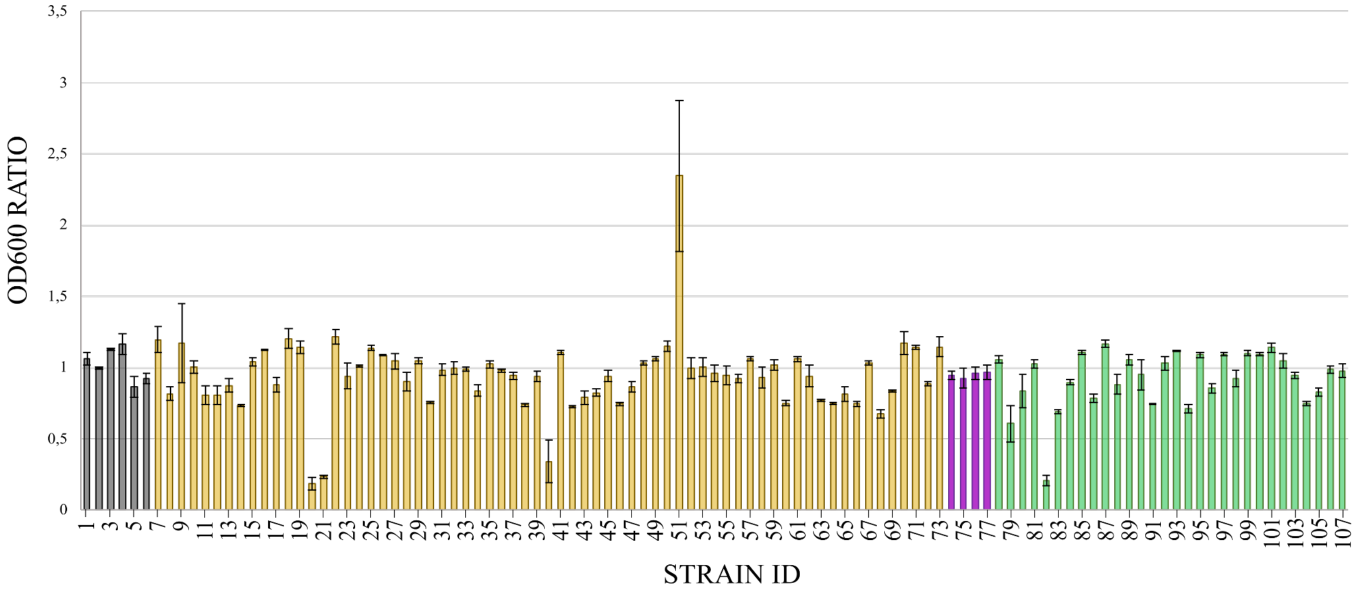
YNB+aa 48h
OD600 RATIO
STRAIN ID

Supplementary Figure 9: Comparison of the growth ratio of strains grouped according to the species. The growth ratio was calculated as the OD of the yeast culture in the tested medium divided by the OD of the yeast culture in YPD (YP supplemented with 2% glucose). The letters on top of the points and boxplots indicate the yeast species showing significantly different growth capabilities compared to the indicated species in the medium reported in the plot label. **A-B)** growth rates in the rich medium YP (1% Yeast extract, 2% Peptone) supplemented with 2% arabinose or **C-D)** supplemented with 2% sucrose; growth rates in the minimum medium YNB (Yeast Nitrogen Base)

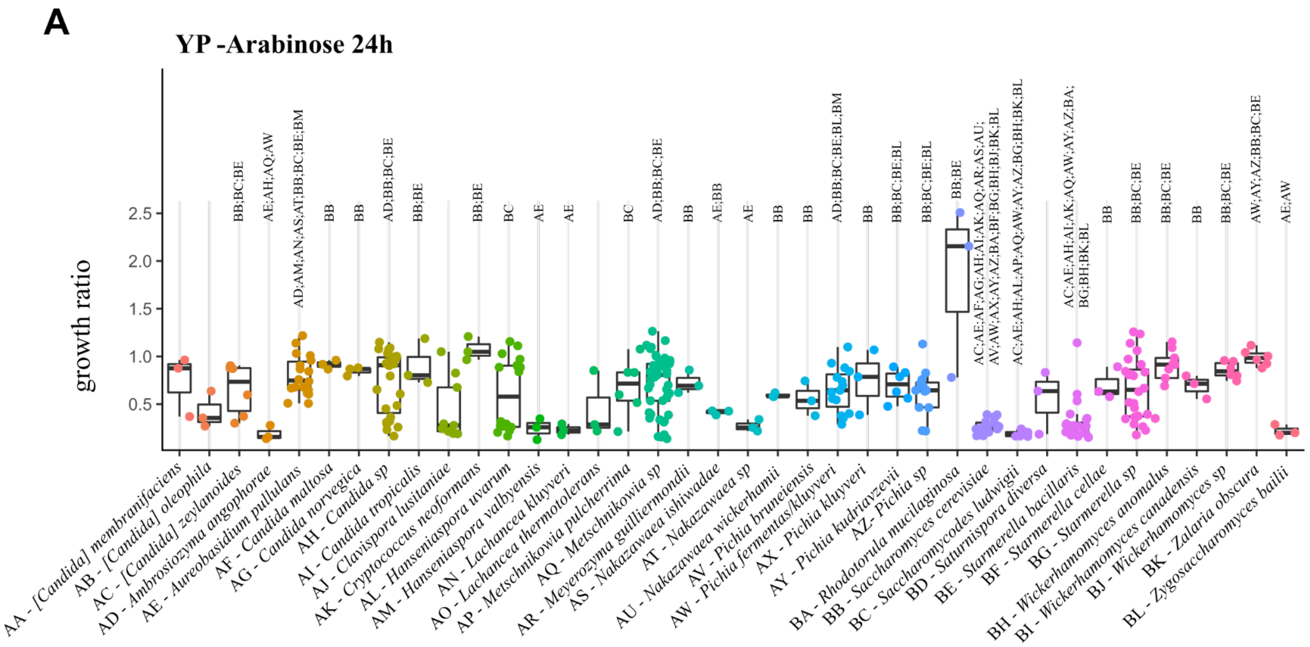

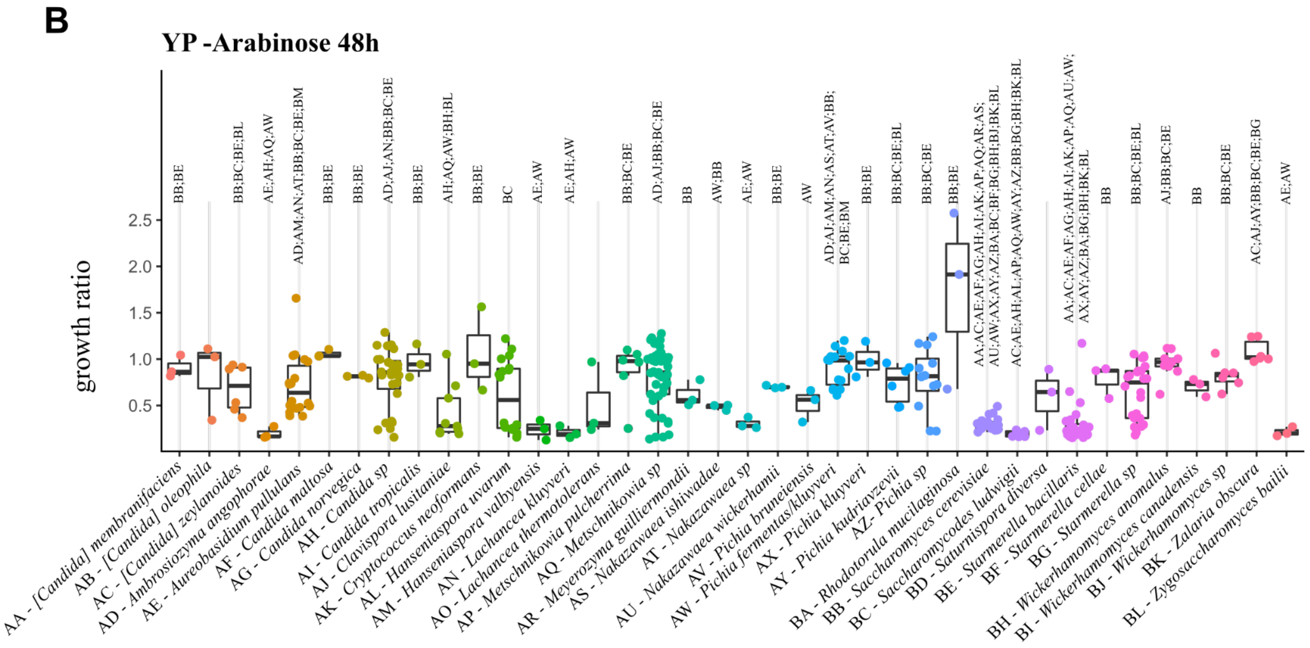

without **(E-F)** and with **(G-H)** the supplementation of amino acids.

C

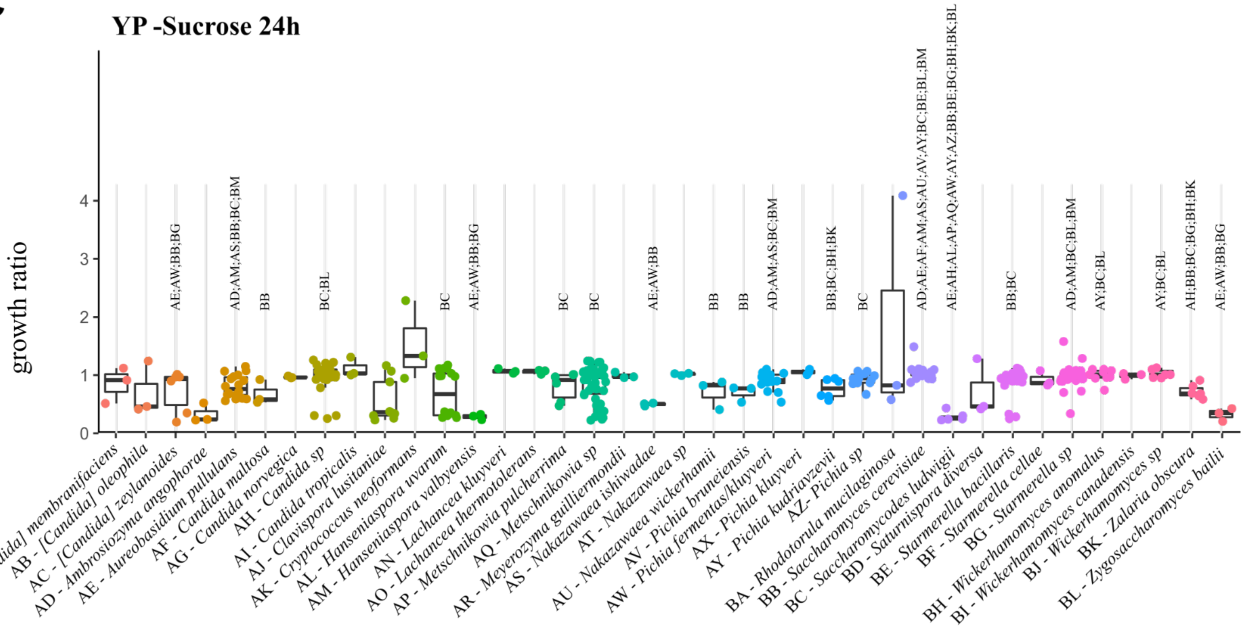

D

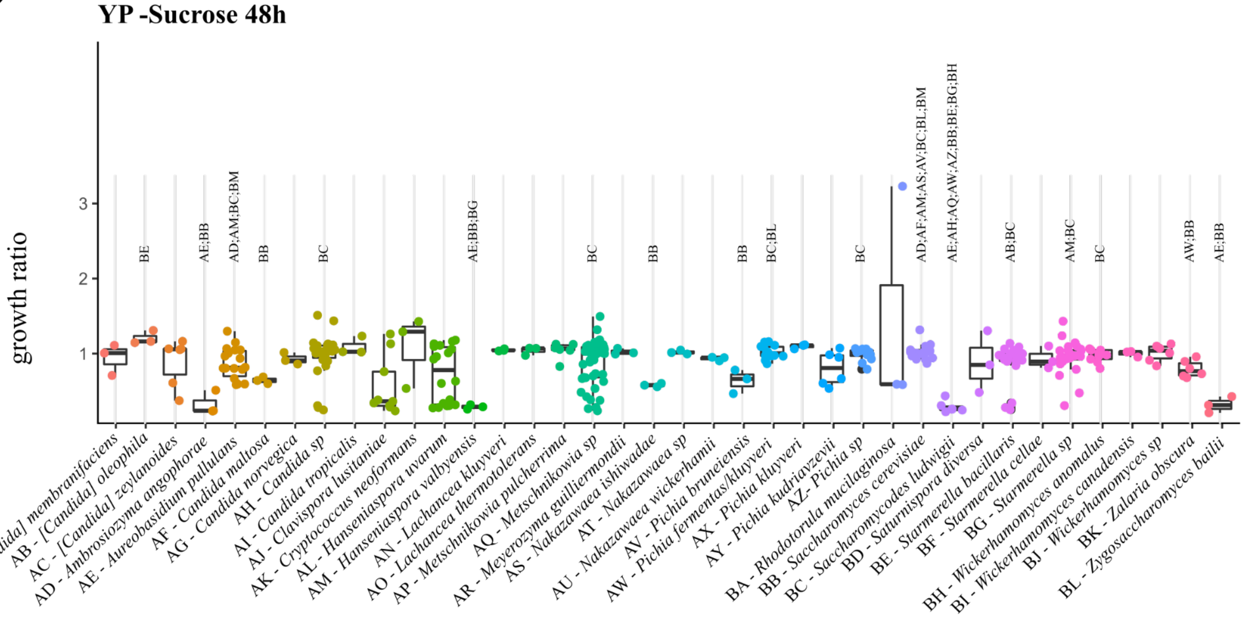

E

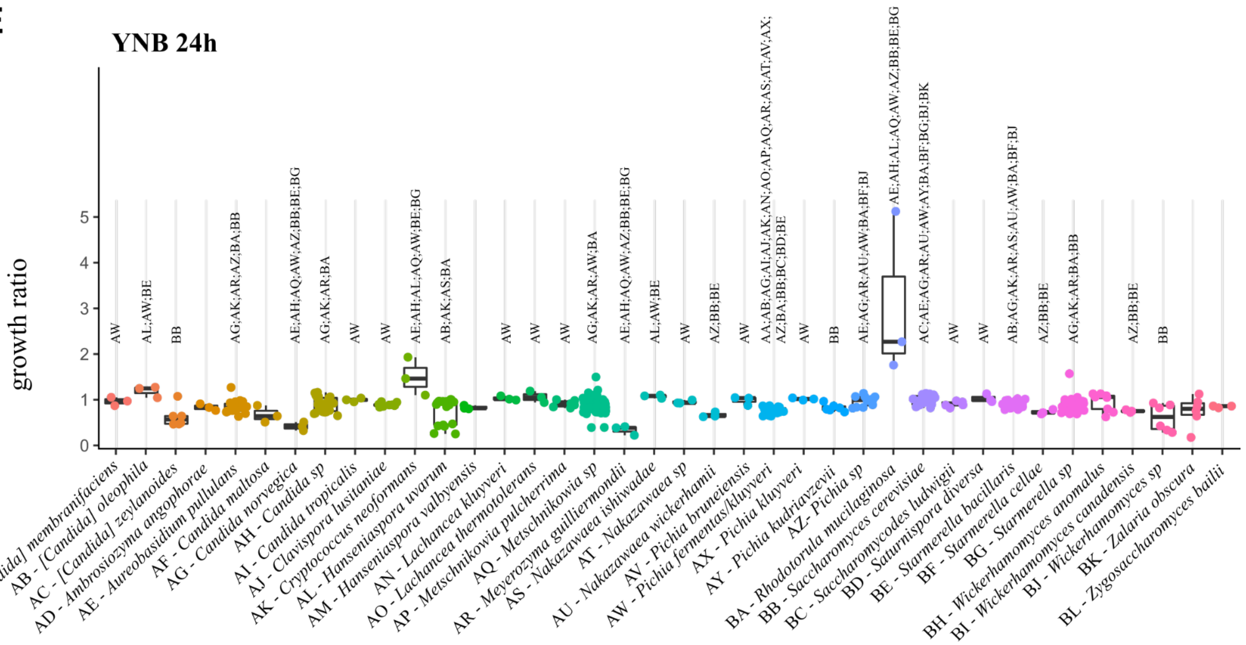
YNB 24h
growth ratio
AA - [Candida] membranifaciens
AB - [Candida] oleophila
AC - [Candida] zeylanoides
AD - Ambrosiozyma angophorae
AE - Aureobasidium pullulans
AF - Candida maltosa
AG - Candida norvegica
AH - Candida sp
AI - Candida tropicalis
AJ - Clavispora lusitaniae
AK - Cryptococcus neoformans
AL - Hanseniaspora uvarum
AM - Hanseniaspora valbyensis
AN - Lachancea kluyveri
AO - Lachancea thermotolerans
AP - Metschnikowia pulcherrima
AQ - Metschnikowia sp
AR - Meyerozyma guilliermondii
AS - Nakazawaea ishiwadae
AT - Nakazawaea sp
AU - Nakazawaea wickerhamii
AV - Pichia bruneiensis
AW - Pichia fermentas/kluyveri
AX - Pichia kluyveri
AY - Pichia kudriavzevii
AZ- Pichia sp
BA - Rhodotorula mucilaginosa
BB - Saccharomyces cerevisiae
BC - Saccharomycodes ludwigii
BD - Saturnispora diversa
BE - Starmerella bacillaris
BF - Starmerella cellae
BG - Starmerella sp
BH - Wickerhamomyces anomalus
BI - Wickerhamomyces canadensis
BJ - Wickerhamomyces sp
BK - Zalaria obscura
BL - Zygosaccharomyces bailii

F

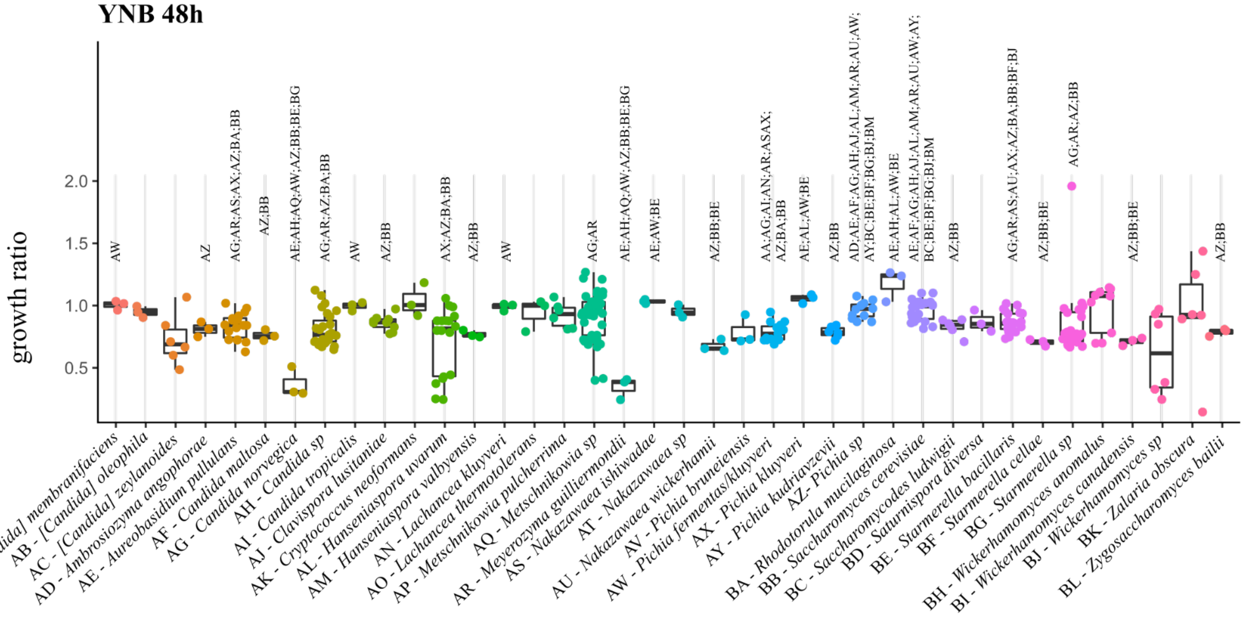
YNB 48h
growth ratio
AA - [Candida] membranifaciens
AB - [Candida] oleophila
AC - [Candida] zeylanoides
AD - Ambrosiozyma angophorae
AE - Aureobasidium pullulans
AF - Candida maltosa
AG - Candida norvegica
AH - Candida sp
AI - Candida tropicalis
AJ - Clavispora lusitaniae
AK - Cryptococcus neoformans
AL - Hanseniaspora uvarum
AM - Hanseniaspora valbyensis
AN - Lachancea kluyveri
AO - Lachancea thermotolerans
AP - Metschnikowia pulcherrima
AQ - Metschnikowia sp
AR - Meyerozyma guilliermondii
AS - Nakazawaea ishiwadae
AT - Nakazawaea sp
AU - Nakazawaea wickerhamii
AV - Pichia bruneiensis
AW - Pichia fermentas/kluyveri
AX - Pichia kluyveri
AY - Pichia kudriavzevii
AZ- Pichia sp
BA - Rhodotorula mucilaginosa
BB - Saccharomyces cerevisiae
BC - Saccharomycodes ludwigii
BD - Saturnispora diversa
BE - Starmerella bacillaris
BF - Starmerella cellae
BG - Starmerella sp
BH - Wickerhamomyces anomalus
BI - Wickerhamomyces canadensis
BJ - Wickerhamomyces sp
BK - Zalaria obscura
BL - Zygosaccharomyces bailii

G

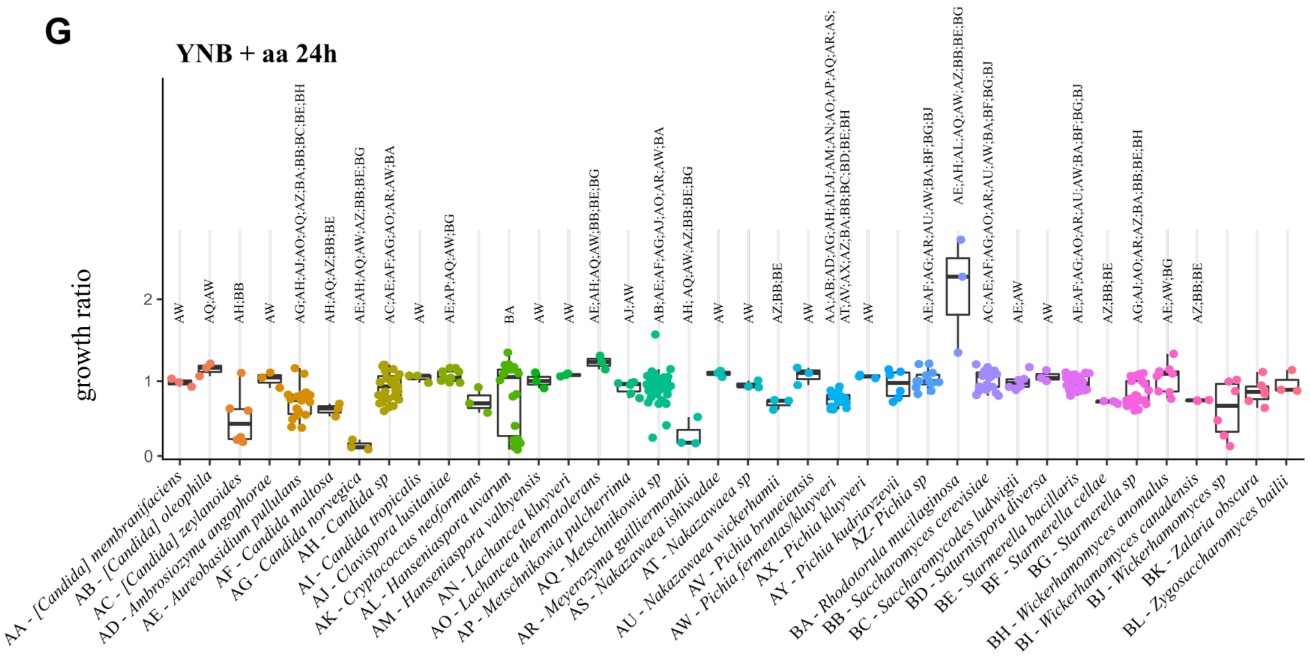
YNB + aa 24h
growth ratio
AA - [Candida] membranifaciens
AB - [Candida] oleophila
AC - [Candida] zeylanoides
AD - Ambrosiozyma angophorae
AE - Aureobasidium pullulans
AF - Candida maltosa
AG - Candida norvegica
AH - Candida sp
AI - Candida tropicalis
AJ - Clavispora lusitaniae
AK - Cryptococcus neoformans
AL - Hanseniaspora uvarum
AM - Hanseniaspora valbyensis
AN - Lachancea kluyveri
AO - Lachancea thermotolerans
AP - Metschnikowia pulcherrima
AQ - Metschnikowia sp
AR - Meyerozyma guilliermondii
AS - Nakazawaea ishiwadae
AT - Nakazawaea sp
AU - Nakazawaea wickerhamii
AV - Pichia bruneiensis
AW - Pichia fermentas/kluyveri
AX - Pichia kluyveri
AY - Pichia kudriavzevii
AZ- Pichia sp
BA - Rhodotorula mucilaginosa
BB - Saccharomyces cerevisiae
BC - Saccharomycodes ludwigii
BD - Saturnispora diversa
BE - Starmerella bacillaris
BF - Starmerella cellae
BG - Starmerella sp
BH - Wickerhamomyces anomalus
BI - Wickerhamomyces canadensis
BJ - Wickerhamomyces sp
BK - Zalaria obscura
BL - Zygosaccharomyces bailii

H

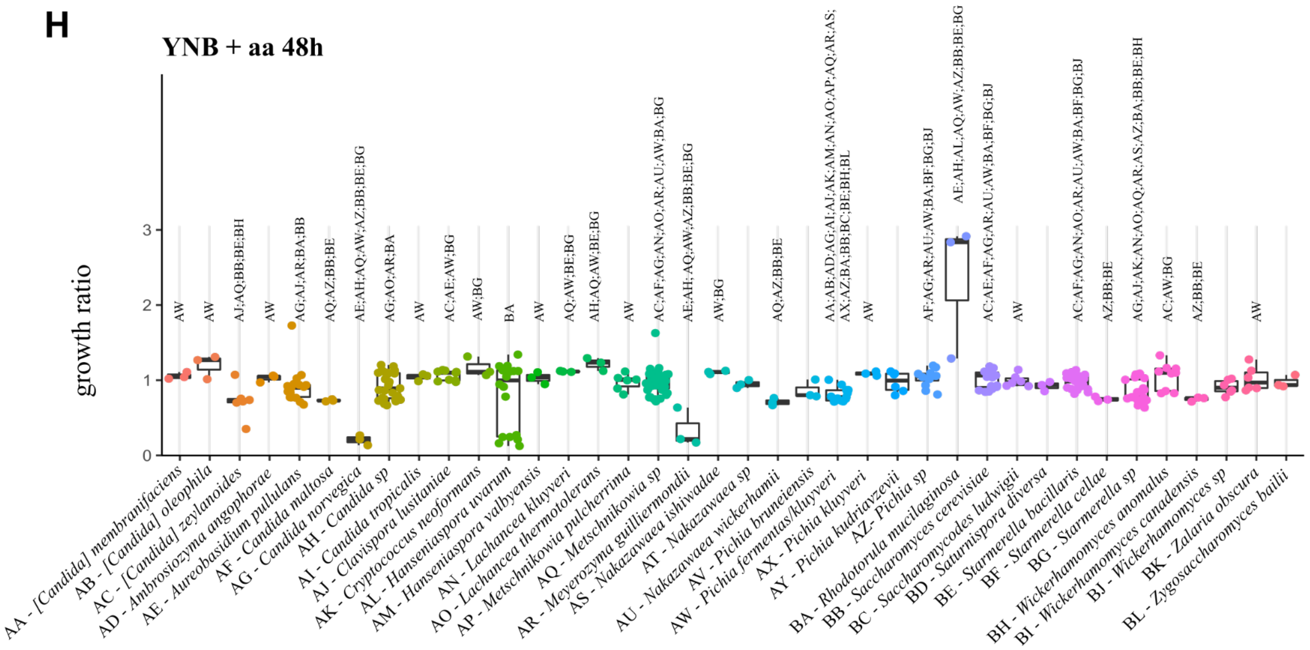
YNB + aa 48h
growth ratio
AA - [Candida] membranifaciens
AB - [Candida] oleophila
AC - [Candida] zeylanoides
AD - Ambrosiozyma angophorae
AE - Aureobasidium pullulans
AF - Candida maltosa
AG - Candida norvegica
AH - Candida sp
AI - Candida tropicalis
AJ - Clavispora lusitaniae
AK - Cryptococcus neoformans
AL - Hanseniaspora uvarum
AM - Hanseniaspora valbyensis
AN - Lachancea kluyveri
AO - Lachancea thermotolerans
AP - Metschnikowia pulcherrima
AQ - Metschnikowia sp
AR - Meyerozyma guilliermondii
AS - Nakazawaea ishiwadae
AT - Nakazawaea sp
AU - Nakazawaea wickerhamii
AV - Pichia bruneiensis
AW - Pichia fermentas/kluyveri
AX - Pichia kluyveri
AY - Pichia kudriavzevii
AZ- Pichia sp
BA - Rhodotorula mucilaginosa
BB - Saccharomyces cerevisiae
BC - Saccharomycodes ludwigii
BD - Saturnispora diversa
BE - Starmerella bacillaris
BF - Starmerella cellae
BG - Starmerella sp
BH - Wickerhamomyces anomalus
BI - Wickerhamomyces canadensis
BJ - Wickerhamomyces sp
BK - Zalaria obscura
BL - Zygosaccharomyces bailii

[Supplementary Figure 10](). **Yeast species clustering according to the quantified traits. A)** Results of Kmeans analysis to select the best number of clusters of samples distributed according to the components of the PCA performed on phenotypic data. **B)** Clustering of tested strains, representative of the species isolated in this study from insects' guts and of strains isolated from wine musts. **C)** Comparison of quantified traits among strains grouped according to the clustering inferred by means of the Kmeans analysis. The numbers on top of the points and boxplots indicate the clusters showing significantly different growth capabilities compared to the indicated cluster in the medium reported in the plot label. yIVC= yeasts isolated from Insects caught in Vineyards used as Controls; yIVW= yeasts isolated from Insects caught in Vineyards close to Woods.

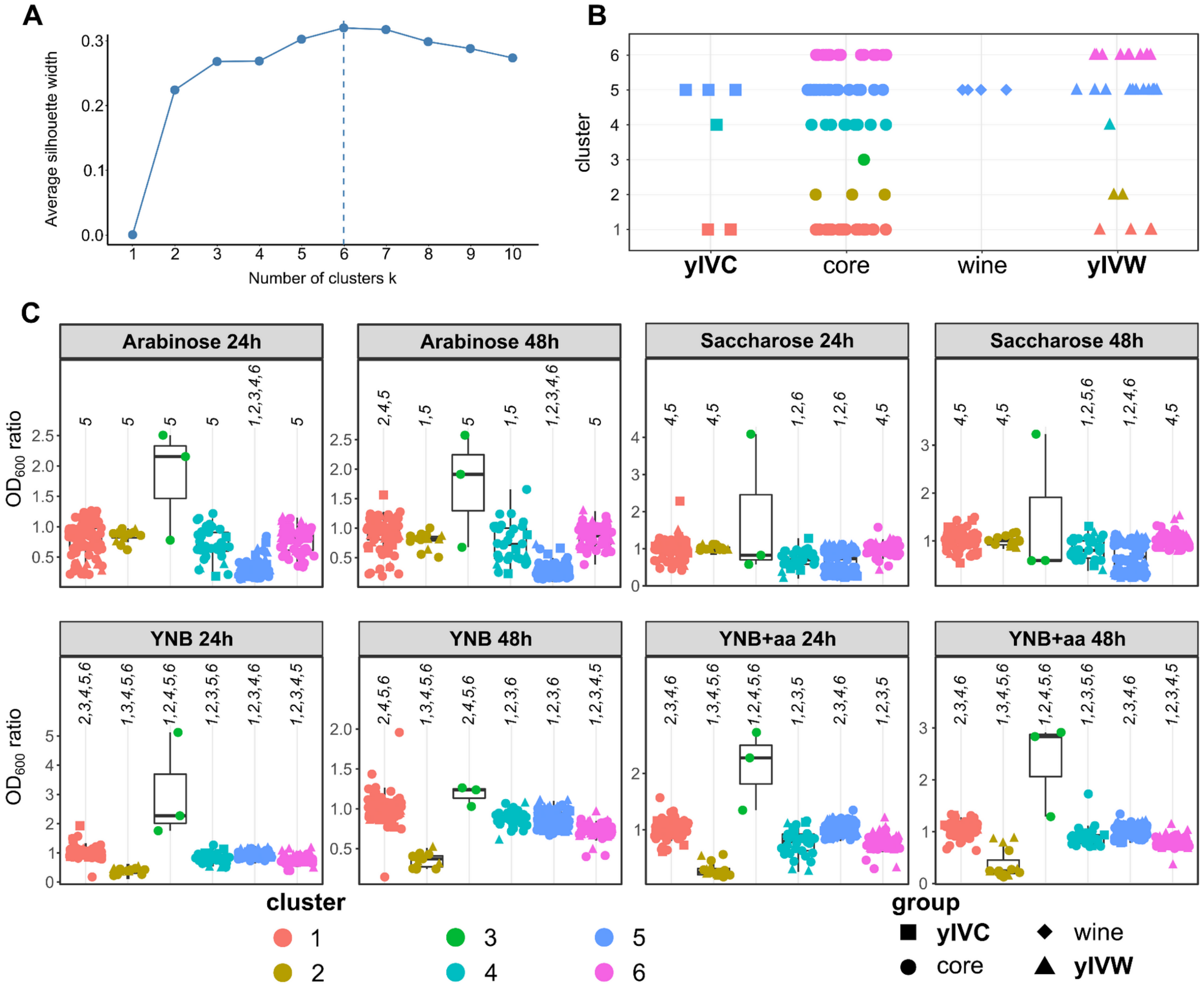

Supplementary Table 6. **Results of Wilcoxon-Mann-Whitney tests on phenotypes of tested strains.** Wilcoxon-Mann-Whitney tests were performed to compare the growth capabilities of strains grouped according to the clustering based on the PCA coordinates (see **Fig. 3B-C** and **Supplementary Table 4**). Only significant results are shown (fdr<0.05).

condition	groupA	groupB	W	p.value	fdr
Arabinose 48h	1	2	922	1.10E-02	2.04E-02
YNB 24h	1	2	1290	1.76E-09	7.91E-09
YNB 48h	1	2	1290	1.76E-09	7.91E-09
YNB+aa 24h	1	2	1305	7.25E-10	3.87E-09
YNB+aa 48h	1	2	1291	1.66E-09	7.91E-09
YNB 24h	1	3	1	3.74E-03	7.37E-03
YNB+aa 24h	1	3	1	3.74E-03	7.37E-03
YNB+aa 48h	1	3	4	4.62E-03	8.88E-03
Arabinose 48h	1	4	1852	1.45E-02	2.64E-02
Sucrose 24h	1	4	2434	4.48E-09	1.79E-08
Sucrose 48h	1	4	2245	1.99E-06	6.09E-06
YNB 24h	1	4	2353	7.06E-08	2.42E-07
YNB 48h	1	4	2385	2.44E-08	8.78E-08
YNB+aa 24h	1	4	2357	6.20E-08	2.18E-07
YNB+aa 48h	1	4	2392	1.92E-08	7.10E-08
Arabinose 24h	1	5	8746	5.06E-25	1.04E-23
Arabinose 48h	1	5	8939	2.63E-27	1.26E-25
Sucrose 24h	1	5	7414	4.16E-12	3.33E-11
Sucrose 48h	1	5	7899	3.09E-16	3.42E-15
YNB 24h	1	5	6511	3.72E-06	1.11E-05
YNB 48h	1	5	7559	2.84E-13	2.72E-12
YNB 24h	1	6	5998	4.05E-20	5.85E-19
YNB 48h	1	6	6358	2.58E-25	7.26E-24

YNB+aa 24h	1	6	5946	2.02E-19	2.65E-18
YNB+aa 48h	1	6	6139	4.44E-22	7.99E-21
Arabinose 48h	2	1	383	1.10E-02	2.04E-02
YNB 24h	2	1	15	1.76E-09	7.91E-09
YNB 48h	2	1	15	1.76E-09	7.91E-09
YNB+aa 24h	2	1	0	7.25E-10	3.87E-09
YNB+aa 48h	2	1	14	1.66E-09	7.91E-09
YNB 24h	2	3	0	2.45E-03	5.69E-03
YNB 48h	2	3	0	2.45E-03	5.69E-03
YNB+aa 24h	2	3	0	2.45E-03	5.69E-03
YNB+aa 48h	2	3	0	2.45E-03	5.69E-03
Sucrose 24h	2	4	463	7.40E-08	2.48E-07
Sucrose 48h	2	4	370	5.70E-03	1.07E-02
YNB 24h	2	4	4	2.20E-11	1.58E-10
YNB 48h	2	4	0	1.83E-12	1.55E-11
YNB+aa 24h	2	4	20	4.92E-09	1.91E-08
YNB+aa 48h	2	4	35	1.33E-07	4.27E-07
Arabinose 24h	2	5	1612	5.84E-10	3.36E-09
Arabinose 48h	2	5	1609	6.76E-10	3.74E-09
Sucrose 24h	2	5	1275	3.31E-04	8.65E-04
Sucrose 48h	2	5	1236	1.01E-03	2.54E-03
YNB 24h	2	5	0	3.93E-10	2.36E-09
YNB 48h	2	5	0	3.93E-10	2.36E-09
YNB+aa 24h	2	5	0	3.93E-10	2.36E-09
YNB+aa 48h	2	5	15	8.22E-10	4.23E-09
YNB 24h	2	6	18	3.87E-09	1.59E-08
YNB 48h	2	6	10	2.28E-09	9.67E-09
YNB+aa 24h	2	6	10	2.28E-09	9.67E-09

YNB+aa 48h	2	6	161	1.42E-05	4.16E-05
YNB 24h	3	1	260	3.74E-03	7.37E-03
YNB+aa 24h	3	1	260	3.74E-03	7.37E-03
YNB+aa 48h	3	1	257	4.62E-03	8.88E-03
YNB 24h	3	2	45	2.45E-03	5.69E-03
YNB 48h	3	2	45	2.45E-03	5.69E-03
YNB+aa 24h	3	2	45	2.45E-03	5.69E-03
YNB+aa 48h	3	2	45	2.45E-03	5.69E-03
YNB 24h	3	4	99	2.80E-04	7.47E-04
YNB 48h	3	4	97	1.12E-03	2.78E-03
YNB+aa 24h	3	4	99	2.80E-04	7.47E-04
YNB+aa 48h	3	4	98	5.60E-04	1.44E-03
Arabinose 24h	3	5	323	3.52E-03	7.33E-03
Arabinose 48h	3	5	323	3.52E-03	7.33E-03
YNB 24h	3	5	324	3.32E-03	7.33E-03
YNB 48h	3	5	320	4.18E-03	8.14E-03
YNB+aa 24h	3	5	323	3.52E-03	7.33E-03
YNB+aa 48h	3	5	323	3.52E-03	7.33E-03
YNB 24h	3	6	225	3.61E-03	7.33E-03
YNB 48h	3	6	225	3.61E-03	7.33E-03
YNB+aa 24h	3	6	225	3.61E-03	7.33E-03
YNB+aa 48h	3	6	225	3.61E-03	7.33E-03
Arabinose 48h	4	1	1019	1.45E-02	2.64E-02
Sucrose 24h	4	1	437	4.48E-09	1.79E-08
Sucrose 48h	4	1	626	1.99E-06	6.09E-06
YNB 24h	4	1	518	7.06E-08	2.42E-07
YNB 48h	4	1	486	2.44E-08	8.78E-08
YNB+aa 24h	4	1	514	6.20E-08	2.18E-07

YNB+aa 48h	4	1	479	1.92E-08	7.10E-08
Sucrose 24h	4	2	32	7.40E-08	2.48E-07
Sucrose 48h	4	2	125	5.70E-03	1.07E-02
YNB 24h	4	2	491	2.20E-11	1.58E-10
YNB 48h	4	2	495	1.83E-12	1.55E-11
YNB+aa 24h	4	2	475	4.92E-09	1.91E-08
YNB+aa 48h	4	2	460	1.33E-07	4.27E-07
YNB 24h	4	3	0	2.80E-04	7.47E-04
YNB 48h	4	3	2	1.12E-03	2.78E-03
YNB+aa 24h	4	3	0	2.80E-04	7.47E-04
YNB+aa 48h	4	3	1	5.60E-04	1.44E-03
Arabinose 24h	4	5	3263	5.63E-13	5.07E-12
Arabinose 48h	4	5	3332	4.52E-14	4.65E-13
Sucrose 48h	4	5	2237	2.69E-02	4.84E-02
YNB 24h	4	5	997	1.33E-04	3.69E-04
YNB+aa 24h	4	5	541	1.54E-09	7.63E-09
YNB+aa 48h	4	5	713	1.96E-07	6.14E-07
Sucrose 24h	4	6	389	1.55E-08	5.88E-08
Sucrose 48h	4	6	593	1.75E-05	5.03E-05
YNB 24h	4	6	1655	5.42E-03	1.03E-02
YNB 48h	4	6	2217	6.60E-11	4.53E-10
YNB+aa 48h	4	6	1825	9.04E-05	2.55E-04
Arabinose 48h	5	1	457	2.63E-27	1.26E-25
Sucrose 24h	5	1	1982	4.16E-12	3.33E-11
Sucrose 48h	5	1	1497	3.09E-16	3.42E-15
YNB 24h	5	1	2885	3.72E-06	1.11E-05
YNB 48h	5	1	1837	2.84E-13	2.72E-12
Arabinose 48h	5	2	11	6.76E-10	3.74E-09

Sucrose 24h	5	2	345	3.31E-04	8.65E-04
Sucrose 48h	5	2	384	1.01E-03	2.54E-03
YNB 24h	5	2	1620	3.93E-10	2.36E-09
YNB 48h	5	2	1620	3.93E-10	2.36E-09
YNB+aa 24h	5	2	1620	3.93E-10	2.36E-09
YNB+aa 48h	5	2	1605	8.22E-10	4.23E-09
Arabinose 48h	5	3	1	3.52E-03	7.33E-03
YNB 24h	5	3	0	3.32E-03	7.33E-03
YNB 48h	5	3	4	4.18E-03	8.14E-03
YNB+aa 24h	5	3	1	3.52E-03	7.33E-03
YNB+aa 48h	5	3	1	3.52E-03	7.33E-03
Arabinose 48h	5	4	232	4.52E-14	4.65E-13
Sucrose 48h	5	4	1327	2.69E-02	4.84E-02
YNB 24h	5	4	2567	1.33E-04	3.69E-04
YNB+aa 24h	5	4	3023	1.54E-09	7.63E-09
YNB+aa 48h	5	4	2851	1.96E-07	6.14E-07
Arabinose 24h	5	6	178	4.49E-28	3.23E-26
Arabinose 48h	5	6	46	6.61E-30	9.52E-28
Sucrose 24h	5	6	2182	1.16E-07	3.81E-07
Sucrose 48h	5	6	1685	1.96E-11	1.48E-10
YNB 24h	5	6	7101	4.89E-18	5.87E-17
YNB 48h	5	6	7288	4.06E-20	5.85E-19
YNB+aa 24h	5	6	7730	1.62E-25	5.82E-24
YNB+aa 48h	5	6	7709	3.02E-25	7.26E-24
YNB 24h	6	1	527	4.05E-20	5.85E-19
YNB 48h	6	1	167	2.58E-25	7.26E-24
YNB+aa 24h	6	1	579	2.02E-19	2.65E-18
YNB+aa 48h	6	1	386	4.44E-22	7.99E-21

YNB 24h	6	2	1107	3.87E-09	1.59E-08
YNB 48h	6	2	1115	2.28E-09	9.67E-09
YNB+aa 24h	6	2	1115	2.28E-09	9.67E-09
YNB+aa 48h	6	2	964	1.42E-05	4.16E-05
YNB 24h	6	3	0	3.61E-03	7.33E-03
YNB 48h	6	3	0	3.61E-03	7.33E-03
YNB+aa 24h	6	3	0	3.61E-03	7.33E-03
YNB+aa 48h	6	3	0	3.61E-03	7.33E-03
Sucrose 24h	6	4	2086	1.55E-08	5.88E-08
Sucrose 48h	6	4	1882	1.75E-05	5.03E-05
YNB 24h	6	4	820	5.42E-03	1.03E-02
YNB 48h	6	4	258	6.60E-11	4.53E-10
YNB+aa 48h	6	4	650	9.04E-05	2.55E-04
Arabinose 24h	6	5	7922	4.49E-28	3.23E-26
Arabinose 48h	6	5	8054	6.61E-30	9.52E-28
Sucrose 24h	6	5	5918	1.16E-07	3.81E-07
Sucrose 48h	6	5	6415	1.96E-11	1.48E-10
YNB 24h	6	5	999	4.89E-18	5.87E-17
YNB 48h	6	5	812	4.06E-20	5.85E-19
YNB+aa 24h	6	5	370	1.62E-25	5.82E-24
YNB+aa 48h	6	5	391	3.02E-25	7.26E-24

Supplementary statistical results

Detailed results of the statistical analyses carried out in the study.

Number of insects

- Comparison of the number of insects caught in spring and harvesting samplings:
  Wilcoxon-Mann-Whitney W=70 p=0.001
- Comparison of the number of insect species caught in Area 1 and Area 2:
  Wilcoxon-Mann-Whitney W=0, fdr=0.026
- Comparison of the number of insect species caught in Area 1 and Area 3:
  Wilcoxon-Mann-Whitney W=0, fdr=0.027

Yeast abundance

- Comparison of the yeast abundance in insects caught in vineyards close to wooded areas and in control vineyards:
  Wilcoxon-Mann-Whitney W=8987.5, p=0.014
- Comparison of the yeast abundance in insects caught in Area 3 and Area 1 vineyards:
  Wilcoxon-Mann-Whitney W= 5171.2, fdr= $7.732e^{-03}$
- Comparison of the yeast abundance in insects caught in Area 3 and Area 2 vineyards:
  Wilcoxon-Mann-Whitney W= 4358.5, fdr= $3.144e^{-05}$
- Comparison of the yeast abundance in *Polistes gallicus* and *Vespa crabro* insects:
  Wilcoxon-Mann-Whitney W=187, fdr=$2.098e^{-04}$
- Comparison of the yeast abundance in *Polistes gallicus* and *Vespula germanica* insects:
  Wilcoxon-Mann-Whitney W=364, fdr=$1.226e^{-03}$
- Comparison of the yeast abundance in *Polistes gallicus* and *Vespula vulgaris* insects:
  Wilcoxon-Mann-Whitney W=102, fdr=$8.272e^{-03}$
- Comparison of the yeast abundance in *Polistes dominulus* and *Vespa crabro* insects:
  Wilcoxon-Mann-Whitney W=569, fdr=$5.849e^{-06}$
- Comparison of the yeast abundance in *Polistes dominulus* and *Vespula germanica* insects:
  Wilcoxon-Mann-Whitney W=1072, fdr=$3.144e^{-05}$
- Comparison of the yeast abundance in *Polistes dominulus* and *Vespula vulgaris* insects:
  Wilcoxon-Mann-Whitney W=264, fdr=$4.423e^{-04}$
- Comparison of the yeast abundance in insects caught in the harvesting and spring samplings:
  Wilcoxon-Mann-Whitney W=3967.5, p=$5.849e^{-06}$

Yeast alpha diversity

- Comparison of the alpha diversity of yeast populations in insects caught in vineyards close to wooded areas (Mean α-diversity=1.44±1.55) and far from wooded areas (Mean α-diversity=1.02±1.36):
  Wilcoxon-Mann-Whitney W=5671.5, p-value=0.017
- Comparison of the alpha diversity of yeast populations in insects caught in spring (Mean α-diversity=0.71±1.05) and harvest 2020 (Mean α-diversity=1.35±1.23):
  Wilcoxon-Mann-Whitney W = 1335.5, p-value = 0.0009
- Comparison of the alpha diversity of yeast populations in insects caught in spring (Mean α-diversity=0.71±1.05) and harvest 2021 (Mean α-diversity=1.58±1.73):
  Wilcoxon-Mann-Whitney W = 2917.5, p-value = 0.0008

- Comparison of the alpha diversity of yeast populations in insects caught in harvest 2020 (Mean α-diversity=1.35±1.23) and harvest 2021 (Mean α-diversity=1.587±1.731): Wilcoxon-Mann-Whitney W = 2928, p-value = 0.880
- Comparison of the alpha diversity of yeast populations in insects caught in Area 1 (Mean α-diversity=1.13±1.25) and Area 2 (Mean α-diversity=0.73±1.07): Wilcoxon-Mann-Whitney W = 3419, p-value = 0.036
- Comparison of the alpha diversity of yeast populations in insects caught in Area 1 (Mean α-diversity=1.13±1.25) and Area 3 (Mean α-diversity=1.82±1.78): Wilcoxon-Mann-Whitney W = 2840, p-value = 0.017
- Comparison of the alpha diversity of yeast populations in insects caught in Area 2 (Mean α-diversity=0.73±1.07) and Area 3 (Mean α-diversity=1.82±1.78): Wilcoxon-Mann-Whitney W = 1849, p-value = 8.176e-05

Comparison of the alpha diversity of yeast populations in insects grouped according to their species.

	Polistes gallicus (Mean α-diversity= 0.91±0.95)	***Polistes nimphus*** (Mean α-diversity= 1.11±1.24)	***Vespa crabro*** (Mean α-diversity= 2.65±1.95)	***Vespula germanica*** (Mean α-diversity= 1.35±1.48)	***Vespula vulgaris*** (Mean α-diversity= 1.82±1.42)
Polistes dominulus (Mean α-diversity= 0.51±0.78)	W= 613 fdr=0.109	W= 910 fdr=0.043	W= 448 fdr= 6.17e-08	W= 1364 fdr= 0.006	W= 270 fdr= 0.001
Polistes gallicus (Mean α-diversity= 0.91±0.95)		W= 378 fdr= 0.830	W= 200 fdr= 0.002	W= 557.5 fdr= 0.537	W= 123 fdr= 0.091
Polistes nimphus (Mean α-diversity= 1.11±1.24)			W= 347 fdr= 0.002	W= 902.5 fdr= 0.774	W= 212 fdr= 0.152
Vespa crabro (Mean α-diversity= 2.65±1.95)				W= 1414 fdr= 0.004	W= 390 fdr= 0.254
Vespula germanica (Mean α-diversity= 1.35±1.48)					W= 373 fdr= 0.290

Yeast beta diversity

- Comparison of the beta diversity of yeast populations in the gut of wasps caught in vineyards close to woods and in vineyards used as controls (permANOVA on Jaccard distances): permANOVA Df= 1, F=1.984, P=0.010

- Comparison of the beta diversity of yeast populations in the gut of different wasps' species (permANOVA on Jaccard distances): permANOVA Df= 5, F=2.310, P=0.001

- Comparison of the beta diversity of yeast populations in the gut of wasps caught in vineyards located in different areas (permANOVA on Jaccard distances): permANOVA Df= 2, F=2.800, P=0.001

- Comparison of the beta diversity of yeast populations in the gut of wasps caught in different sampling seasons - spring and harvesting (permANOVA on Jaccard distances): permANOVA Df= 1, F=7.952, P=0.001

- Comparison of the beta diversity of yeast populations in the gut of wasps caught in different sampling years (permANOVA on Jaccard distances): permANOVA Df= 1, F=3.120, P=0.001

- Comparison of the beta diversity of yeast populations in the gut of wasps caught in vineyards close to woods and in vineyards used as controls (permANOVA on Unifrac distances): permANOVA Df= 1, F=2.088, P=0.011

- Comparison of the beta diversity of yeast populations in the gut of different wasps' species (permANOVA on Unifrac distances): permANOVA Df= 5, F=2.651, P=0.001

- Comparison of the beta diversity of yeast populations in the gut of wasps caught in vineyards located in different areas (permANOVA on Unifrac distances): permANOVA Df= 2, F=3.420, P=0.001

- Comparison of the beta diversity of yeast populations in the gut of wasps caught in different sampling seasons - spring and harvesting (permANOVA on Unifrac distances): permANOVA Df= 1, F=9.562, P=0.001

- Comparison of the beta diversity of yeast populations in the gut of wasps caught in different sampling years (permANOVA on Unifrac distances): permANOVA Df= 1, F=2.735, P=0.002
